# Novel Non-Peptide Inhibitors against SmCL1 of *Schistosoma mansoni*: In Silico Elucidation, Implications and Evaluation via Knowledge Based Drug Discovery

**DOI:** 10.1371/journal.pone.0123996

**Published:** 2015-05-01

**Authors:** Atif Zafar, Sabahuddin Ahmad, Asim Rizvi, Masood Ahmad

**Affiliations:** 1 Department of Biochemistry, Faculty of Life Sciences, Aligarh Muslim University, Aligarh 202002, Uttar Pradesh, India; 2 Department of Computer Science, Faculty of Natural Sciences, Jamia Millia Islamia, New Delhi 110025, India; University of Edinburgh, UNITED KINGDOM

## Abstract

Schistosomiasis is a major endemic disease known for excessive mortality and morbidity in developing countries. Because praziquantel is the only drug available for its treatment, the risk of drug resistance emphasizes the need to discover new drugs for this disease. Cathepsin SmCL1 is the critical target for drug design due to its essential role in the digestion of host proteins for growth and development of *Schistosoma mansoni*. Inhibiting the function of SmCL1 could control the wide spread of infections caused by *S*. *mansoni* in humans. With this objective, a homology modeling approach was used to obtain theoretical three-dimensional (3D) structure of SmCL1. In order to find the potential inhibitors of SmCL1, a plethora of *in silico* techniques were employed to screen non-peptide inhibitors against SmCL1 via structure-based drug discovery protocol. Receiver operating characteristic (ROC) curve analysis and molecular dynamics (MD) simulation were performed on the results of docked protein-ligand complexes to identify top ranking molecules against the modelled 3D structure of SmCL1. MD simulation results suggest the phytochemical *Simalikalactone-D* as a potential lead against SmCL1, whose pharmacophore model may be useful for future screening of potential drug molecules. To conclude, this is the first report to discuss the virtual screening of non-peptide inhibitors against SmCL1 of *S*. *mansoni*, with significant therapeutic potential. Results presented herein provide a valuable contribution to identify the significant leads and further derivatize them to suitable drug candidates for antischistosomal therapy.

## Introduction

Schistosomiasis (bilharzia) is one of the major health problems of the world, caused by the trematode blood fluke of the genus *Schistosoma*. Report states that the disease infects broad range of humans (391–587 million) in 74 developing countries in tropical and subtropical regions [1]. The causative agents of the schistosomiasis primarily include three species, namely *Schistosoma mansoni*, *S*. *haematobium* and *S*. *japonicum* [2]. In addition, schistosomiasis burden is estimated to exceed 70 million disability-adjusted life-years (DALYS) [3].

Schistosomes undergo several morphological and physiological changes, perpetuating their life cycle between definitive-vertebrate and intermediate-snail hosts. The complex life cycle of schistosomes involves the infective aquatic stage (cercariae), which invade the host skin and transform into schistosomula [4]. Schistosomula travel to the lungs via venous circulation in 4–6 days post penetration and then migrate to the hepatic portal circulation. At this site, the parasites mature and copulate to produce numerous eggs [5]. Studies suggest that human schistosomiasis-associated morbidity results from the immunological reactions in response to the disposition of eggs in the liver and other sites [6]. The complex developmental stages of schistosomes, thus, make it difficult to perform the *in vivo* experiments related to the drug action against these parasites in humans.

In the schistosome gut, cathepsin SmCL1 is located in the gastrodermal cells lining the cecum of the parasite [7]. Here it plays a digestive role by degrading the host haemoglobin which is the main nutrient source for the adult schistosomes [8]. Thus, the important role of cathepsin SmCL1 in the metabolism of the schistosome renders it to be a crucial target for novel anti-schistosome chemotherapy and immuno-prophylaxis [9,10].

Despite substantial efforts in the past, no effective vaccine has been developed against schistosomiasis. Treatment of schistosomiasis relies only on a single drug, praziquantel [11]. However, the intensive use of praziquantel is an increasing concern as it may lead to the development of drug-resistant strains [12]. Hence, it is prudent to identify anti-schistosomal drugs and new schistosomal protein targets for the control and treatment of this ‘neglected tropical disease’ [13, 14]. In a previous study, it was reported that treating *S*. *mansoni* infected mice with broad spectrum peptide-based cysteine protease inhibitors not only reduced worm burden but also inhibited worm fecundity [15]. This shows that cysteine proteases are potential targets of anti-schistosomal drugs. This finding paves the way for the rescuing of more molecules against cathepsin SmCL1, a utility in prophylactic and therapeutic interventions.

Efforts have been made to identify new cathepsin SmCL1 inhibitors as an alternative to traditional therapy in drug-resistant organisms. Inhibitors such as peptidyl fluoromethyl ketones [15], peptidyl diazomethyl ketones [16], vinyl sulphones [17] and epoxysuccinyl derivatives [7] have been categorised as peptide-based inhibitors of SmCL1. To date, a lot of peptide-based inhibitors of cathepsin SmCL1 have been synthesised and evaluated as a potential cysteine protease targets. However, *in vivo* efficacy of peptide-based inhibitors has been limited due to various pharmacological constraints: solubility, stability and selectivity. Hence, the discovery and optimisation of non-peptide inhibitors is necessary to overcome these limitations for reliable and safer chemotherapeutic treatments [18].

In view of the above facts, SmCL1 was taken as a potential target for the present work. Since the three-dimensional (3D) structure for SmCL1 is yet unavailable, a theoretical 3D structure of SmCL1 was developed using reliable templates via homology modeling protocol. Computational approaches such as molecular docking, virtual screening and MD simulations were carried out to identify novel non-peptide inhibitors against SmCL1. It is expected that the non-peptide phytochemical inhibitors can serve as an alternative to cope up with the limitation of *in vivo* efficacy of peptide inhibitors, and are likely to be developed as potential inhibitors against SmCL1.

## Materials and Methods

### Sequence analysis

The 319 amino acid (aa) long protein sequence of cathepsin SmCL1 of *S*. *mansoni* was retrieved from the universal protein resource (UniProt) database (ID: Q26534) in FASTA format. Based on reported literature, mature sequence of SmCL1 was 215aa long and started from the amino acid residue Ile105 [16]. The physio-chemical properties of SmCL1, such as theoretical isoelectric point (p*I*), molecular weight, total number of positive (+R) and negative (-R) residues, extinction coefficient (EC) [19], instability index (II) [20], aliphatic index [21] and grand average hydropathy (GRAVY) [22] were evaluated by Expasy’s ProtParam server [23]. SOPMA [24] was employed for calculating the secondary structural features of the SmCL1 sequence used in this work.

The mature part of SmCL1 sequence was cleaved off and taken as input for performing BLAST against Protein Data Bank (PDB), using protein-protein BLAST (blastp) [25]. Among the list of homologous sequences identified with the BLAST program, the hits which meet the following criteria were selected as template structure for homology modeling of SmCL1: (1) E-value below 10^–5^; (2) query coverage >95% and sequence identity >35% or query coverage >90% and sequence identity >40%; (3) PDB structure with a resolution < 2.5 Å; (4) PDB structure bound with ligand. The templates and the target sequence (mature SmCL1) were then aligned using the PSI-Coffee mode of T-Coffee (v.9.03) server [26] to give a clear description and relation between the target sequence and the templates considered in this experiment.

### Homology modeling

The 3D structure of SmCL1 was constructed using the ‘Python’ based automodel routine of Modeller (V.9.12) [27]. Two important inputs for Modeller are ‘alignment files in PIR format’ and the ‘atom files’; atom file contains the coordinates of the proteins taken as templates. To obtain a PIR format alignment file, multiple sequence alignment (MSA) using ClustalW [28] was performed between the target sequence and the template sequences. The SmCL1 models were constructed based on different alignments, single and multiple. For each alignment, 100 theoretical protein models were constructed using Modeller.

To validate the above models and to pick a single and best model to proceed with the experiments, a series of model evaluation techniques were incorporated in this study. Starting with the inbuilt routine of Modeller, called normalized DOPE score, Z-score of each model was evaluated. The models with the least scores (Z ≤ -1) were selected for further validations. The servers like ProSA-Web [29, 30], PROCHECK [31], ProQ [32], QMEAN [33] and locally installed TM-score [34] were used to validate the ‘selected’ models and finalize from them a single and best theoretical 3D model of SmCL1.

### Ligand Library Preparation

Since the aim of this study was to identify novel non-peptide inhibitors and to perform comparative analysis of the action of ‘phytochemicals’ on cathepsin SmCL1 with respect to non-phytochemical drugs, we used two separate libraries of ligands for molecular docking. These libraries were prepared using ‘ZINC Database’ and ‘Dr. Duke’s Phytochemical and Ethanobotanical Database’.

- *Ligand library of orally active Drug-like compounds from ZINC Database*: As on March 30^th^ 2014, the ZINC database (http://zinc.docking.org/) [35] had about 35 million purchasable compounds, with an exception of few non-purchasable compounds too. Using the ‘Combination Search’ string of the database, the molecules of the interest were retrieved. While performing combination search, the following parameters were included: (1) Molecular weights 150–500; (2) Octanol–water partition coefficients (log P) ≤5; (3) Rotatable bonds ≤10; (4) Polar surface area ≤150 Å^2^; (5) Hydrogen bond acceptors ≤10; (6) Hydrogen bond donors ≤10; (7) Net charge ≤5. These parameters are basically constituents of Lipinski ‘rule of five’ for a drug to be orally active. Thus, it was ensured that the molecules to be retrieved are ‘drug-like’ and ‘purchasable’, so that the wet lab researchers may proceed with the ‘real’ experiments. Applying the above filters, there were 200 molecules which have shown to inhibit the enzyme ‘Cysteine Protease’ of Eukaryotes. As stated earlier, studies suggest that non-peptide inhibitors could be better against the parasites [18], therefore a manual protocol was adopted in further filtering of non-peptide inhibitors from peptide ones. Later, only those molecules were retrieved which inhibit the cysteine protease of the parasites. This step was repeated to minimize the human error. Finally, 60 compounds were confirmed as non-peptide anti-parasitic inhibitors, and were included in this library for further docking procedures.
- *Ligand library of orally active Drug-like Phytochemicals*: This library included the molecules from the Dr. Duke’s Phytochemical and Ethanobotanical Database (http://ars-grin.gov/duke/). This database was searched and 545 molecules were retrieved on April 2^nd^_,_ 2014 showing the antimalarial, antileschmanial and antitrypanosomic activities. This search criterion was taken into consideration, because the important amino acid residues of SmCL1 of *S*. *mansoni* are closely related to the residues of cysteine proteases of *Plasmodium falciparum*, *Leishmania donovani* and *Trypanosoma cruzi*, which cause important parasitic diseases. The PASS server (http://www.pharmaexpert.ru/passonline/) [36], meant for prediction of activity spectra for substances, was used to screen the retrieved molecules for anti-parasitic activity. The molecules with Pa > 0.7 are likely to exhibit the activity in the experiment. Out of 545, only 167 molecules fall under this criterion and were subjected to further filtration. Applying the filter of drug-likeness (Lipinski ‘rule of five’) using the server Chemicalize.org (http://www.chemicalize.org/) [37], only 55 molecules were left in this library which were further taken for docking procedures.

### Docking studies

All docking studies were performed using the standard AutoDock (v4.2) [38] suit incorporated in MGL tools (v1.5.6) [39], utilising the default protocols of this program. The target protein was prepared using standard protocol and saved into ‘PDBQT’ format using MGL tools. Similarly, before starting the docking protocol, individual ligands from the respective libraries were prepared using the python script ‘prepare_ligand4.py’ present in MGL tools and saved into ‘PDBQT’ format too. The input ‘grid parameter’ files were modified and the grid size was adjusted to 70 points in XYZ dimensions respectively, with default spacing of 0.375 Å, to cover the catalytic region of the target protein SmCL1. Rest all the docking parameters were set to default values.

**Fig 1:**
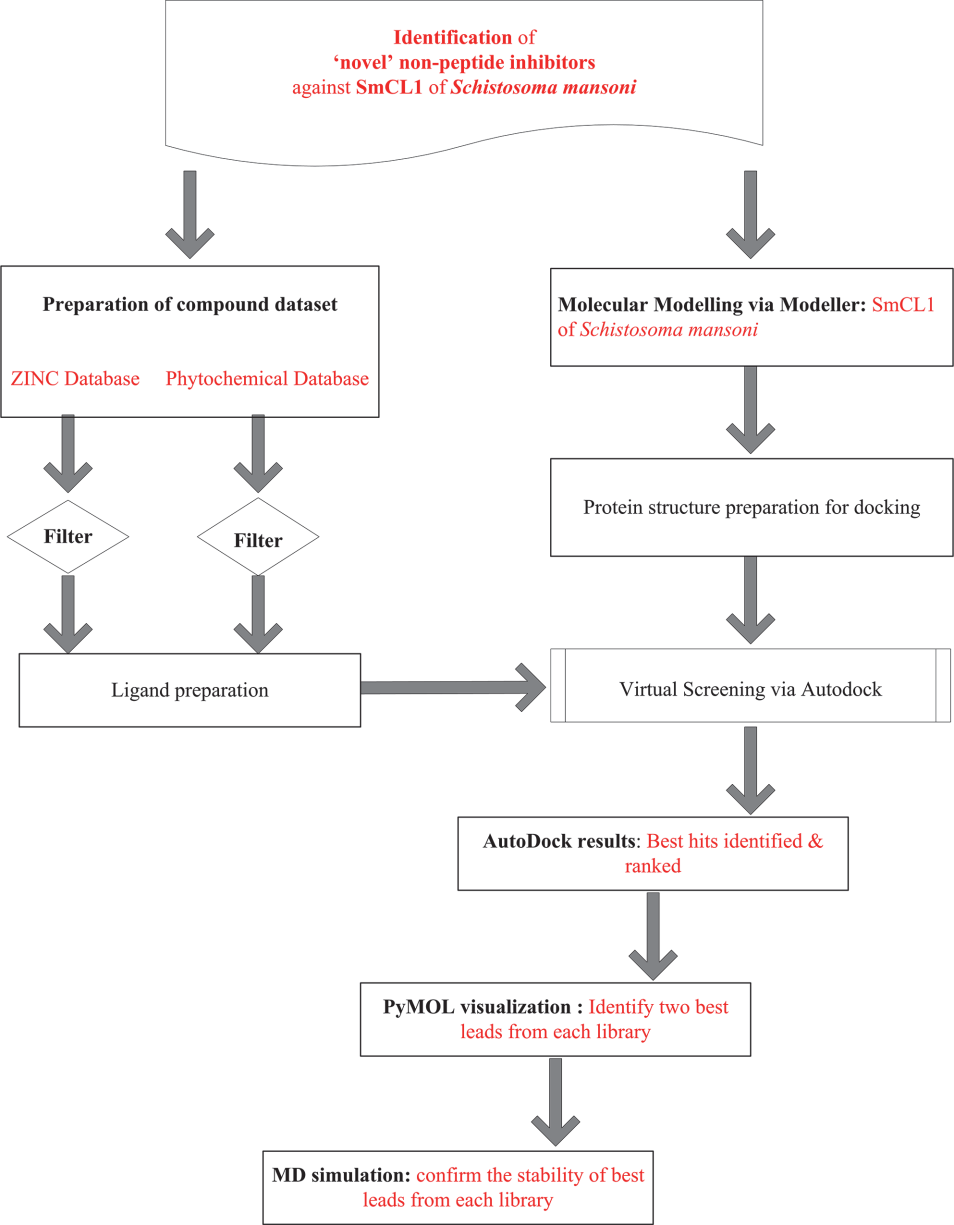
Workflow diagram followed in the *in silico* virtual screening for inhibitors of SmCL1 of *Schistosoma mansoni*.

Top pose, from all the 10 confirmations from AutoDock, was saved as a complex in default ‘PDBQT’ format. The molecules were visualized and written to PDB format using Chimera (v1.8.1) [40]. Hydrogen bond interactions and their distances between the protein and ligands were visualized and measured using PyMOL software [41] (PyMOL Molecular Graphics System, version1.5.0.1, Schrodinger, LLC). The workflow used for the virtual screening is depicted in Fig 1.

### ROC analysis of orally active-drug like compounds in two libraries

To determine the reliability of phytochemical as well as that of ZINC leads obtained, ROC curves [42] for actives and decoys from the two libraries were computed. The ligands which had interaction with important residues of SmCL1 were considered as actives and labeled as 1, while rest were referred to as decoys and labeled as 0. Regarding the ROC curves, it may be noted that a straight line indicates that the model gives no preference to true inhibitors over decoys or vice versa, and the bending more towards left shows a greater accuracy of the model. Simply speaking, the curve bowing more towards left is appreciable and indicates higher ratio of true positives to false positives.

### Stability evaluation of the docked SmCL1 via MD simulations

In order to determine the stability of top docking pose of the best ligands from two libraries, MD simulations were performed using the GROningen MAchine for Chemical Simulations (GROMACS) v4.6.5 package [43].

One known limitation of GROMACS is to parameterize heteroatom groups from PDB files of protein-ligand complex. Hence, in the first step, PDB file of the protein-ligand complex was separated into PDB files of protein and ligand.

The system topology of separated PDB files was prepared individually in two different steps. The ligand topology was prepared using PDB file of ligand over PRODRG server [44]. Protein topology was prepared using PDB of protein with ‘pdb2gmx’ using ‘GROMOS96 43a1 force field’ [45]. Next, ‘unit cell’ was defined and system was filled with water. The complex of protein and ligand was confined into a cubic box maintaining a minimum of 10 Å between the box edges and its surface, while keeping it centered within the box. The resulting system was then solvated with simple point charge (SPC) 216 water model [46]. At physiological pH, the system was found to have a net charge of -8.0. Therefore, counter ions (8Na+) were added to neutralize the system that replaced water molecules at positions of favourable electrostatic potential.

The solvated system was then minimized in 50,000 steps using the steepest descent method, to remove close van der waals contacts. After energy minimization, position restraint dynamics (equilibration run) was performed for 100 picoseconds (ps) (50,000 steps) in two consecutive steps, NVT (Number of particles, Volume and Temperature) equilibration and NPT (Number of particles, Pressure and Temperature) equilibration. To avoid unnecessary distortion of structures during simulations, NVT equilibration was performed, in which all heavy atoms of the protein along with counter ions were restrained to their starting positions and to make sure that water molecules settle (soak) around the structures. The next equilibration was run under NPT conditions to keep the pressure and temperature constant.

Soon after the system was equilibrated, a 2 nanoseconds (ns) long production simulation (MD run) was piloted with a 2 femtoseconds (fs) time step at a pressure of 1 bar, and a temperature of 300 K, to confirm stability of the given system. The interaction energy and intermolecular hydrogen bonds of the system were calculated via the modules g_energy and g_hbond, respectively. The trajectories of simulations were plotted using Gnuplot (v4.6) (http://sourceforge.net/projects/gnuplot).

### Structure-based Pharmacophore model generation via Ligand Scout

The potential SmCL1-inhibitor complex was considered to generate structure-based pharmacophore model via ligand scout (v3.12) [47]. An advanced alignment algorithm in the software allows to overlay and identify pharmacophoric points/features such as hydrogen bond acceptor (HBA), hydrogen bond donor (HBD), ring aromatic (RA), hydrophobic groups (HYP) and hydrogen bonding vectors on ligand to generate the pharmacophore model of potential inhibitor against SmCL1.

## Results and Discussion

The physio-chemical and structural properties of SmCL1 are shown in Table 1. The calculated p*I* (isoelectric point) of SmCL1 is 5.06 (p*I* < 7), which suggests that the protein is acidic in nature. Extinction coefficient (EC) of the protein, which is used to determine the protein-protein and protein-ligand interaction in the medium, was elucidated as 66,265 M^-1^ cm^-1^. The stability of the protein is determined by its instability index (II); with a value less than 40 indicates a stable protein. Instability index of SmCL1 was calculated as 25.03, which shows that it is a stable protein. The aliphatic index (AI), which is regarded as a positive factor for the increase of thermostability of globular proteins, was found to be 76.60. GRAVY value for a protein is an index of the solubility of the proteins in the solution. Its positive and negative values indicate the hydrophobic and hydrophilic nature of the protein in the solution, respectively. GRAVY indices of SmCL1 were found to be -0.267, indicating its hydrophilic nature, and thus showing better interaction with water. The secondary structure of the protein was predicted by the self-optimised prediction method with alignment (SOPMA), which correctly predicts 69.5% of amino acids for a three state description of the secondary structure in alpha helix, beta sheet and random coil.

**Table 1:** Physio-chemical and structural properties of SmCL1.

Physio-chemical Parameters		Secondary Structure Parameters (by SOPMA)	
Property	Analysis	Property	Analysis (%)
Molecular weight	24113.18	Alpha helix	20.93
Isoelectric point	5.06	3_10_ helix	0.00
Positive residues	16	Pi helix	0.00
Negative residues	24	Beta bridge	0.00
Extinction coefficient	66,265	Extended strand	21.86
Instability index	25.03	Beta turn	9.77
Aliphatic index	76.60	Bend region	0.00
GRAVY	-0.267	Random coil	47.44
-	-	Ambiguous states	0.00

Growth, maturation and fecundity of schistosome parasite rely on the ingestion of degraded haemoglobin and serum proteins by SmCL1 [48, 49]. This essential role of SmCL1 in the adult schistosome parasite makes it a suitable target for structure-based drug design. The atomic coordinates of SmCL1 were not available in PDB; therefore homology modeling protocol was necessary to develop a protein model. ModellerV.9.12 program was used to compute homology modeling based on the satisfaction of spatial restraints of the target sequence, which is aligned with the 3D structure of the templates [27].

For the accuracy of homology modeling, it is important to consider the templates whose similarity and identity is close to the target sequence. In this quest, template search and sequence alignments are the crucial steps before starting with the modeling protocol. Therefore, SmCL1 protein sequence was used as a query in a protein-protein BLAST (blastp) search against PDB. Among the best hits, only those 3D templates were retrieved, which satisfy the criteria (1)-(4) (described in materials and methods). Six templates, namely 1EWP, 2P7U, 2XU3, 1S4V, 2FO5 and 1M6D (1M6D and 2FO5 had highest and least blast score, respectively), were finally considered for homology modeling. The detailed information about these templates is given in Table 2.

**Table 2:** Identified homologs of SmCL1 based on the filter parameters discussed in the text.

PDB ID	Chain ID	UniProt ID	Organism	Identity (%)	Coverage (%)	PDB Resolution (Å)	E-value
1EWP	A	P25779	*Trypanosoma cruzi*	45	99	1.75	2.00E-57
1M6D	B	Q9UBX1	*Homo sapiens*	58	99	1.7	7.00E-83
1S4V	B	O65039	*Ricinus communis*	45	97	2.0	2.00E-52
2FO5	A	P25250	*Hordeum vulgare*	45	94	2.2	3.00E-51
2P7U	A	Q95PM0	*Trypanosoma brucei rhodesiense*	44	99	1.65	3.00E-57
2XU3	A	P07711	*Homo sapiens*	44	99	0.9	3.00E-53

**Fig 2:**
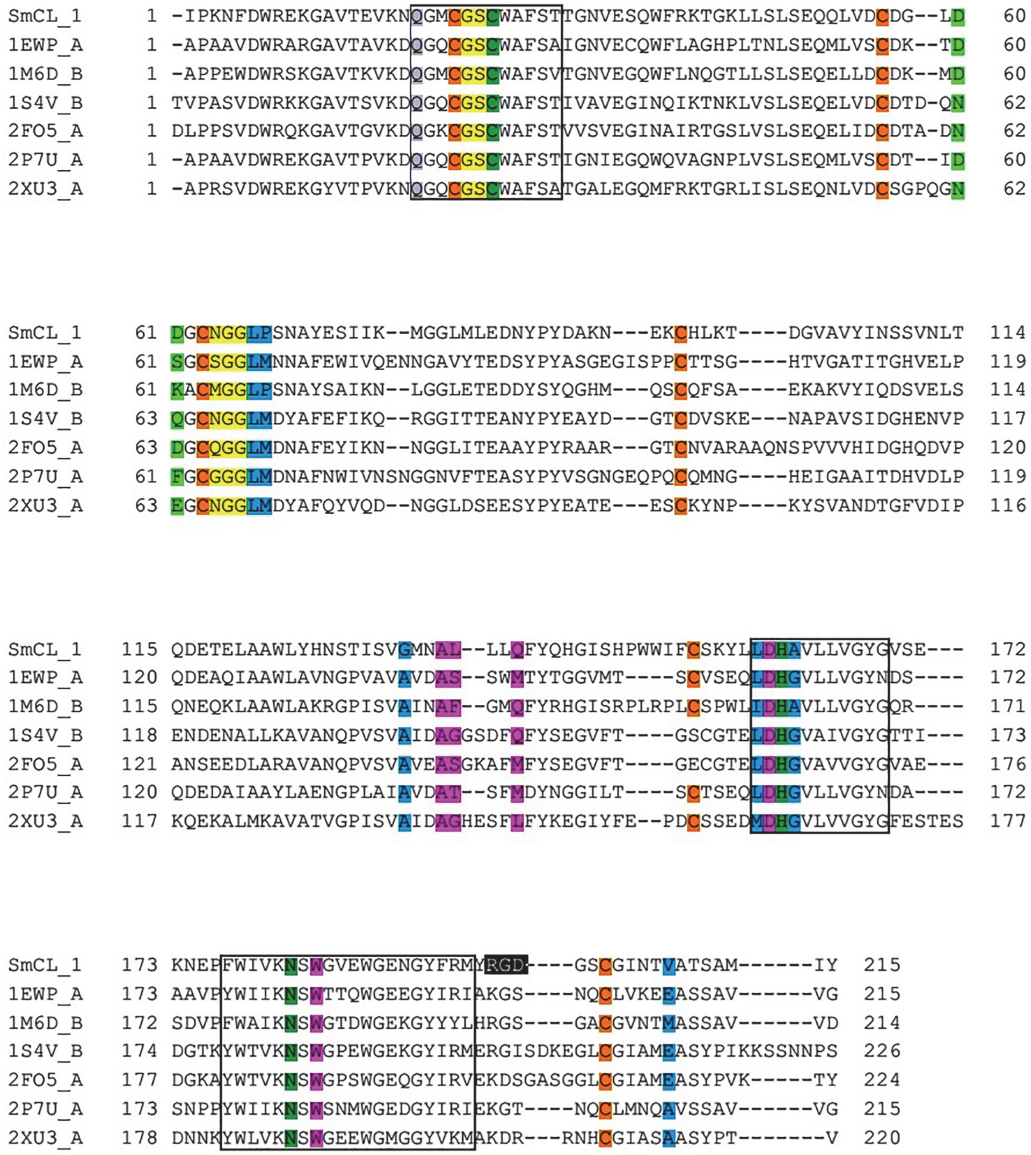
Multiple sequence alignment of the protein sequence SmCL1 and other six templates selected. The *boxes* indicate the conserved cysteine, histidine and asparagine catalytic regions. The catalytic residues forming the triad: Cys25, His161 and Asn182 are highlighted dark green. Glutamine (Gln19) involved in the formation of oxyanion hole is highlighted purple. The residues highlighted orange are cysteine residues forming disulphide bonds. The residues highlighted with blue, yellow, pink and light green correspond to S2, S1, S1’ and S3 pockets, respectively. RGD binding motif of SmCL1 is highlighted in black.

Average sequence homology of SmCL1 with six homologs considered was around 47%, ranging from 44% to 58%. The mature sequence of SmCL1 starting from isoleucine (Ile105) and the sequences of selected templates were subjected to multiple sequence alignment as presented in Fig 2. Alignment confirms the fact that important residues such as catalytic triad: Cys25, His161 and Asn182 (Fig 2), oxyanion hole: Gln19 (Fig 2) and S2 pocket residues (Fig 2) are conserved, and show low polymorphism in the surrounding area (shown in box in Fig 2) of the protein sequence of the selected templates and target protein SmCL1, thus promoting the suitability to use the selected templates for homology modeling of SmCL1.

Generally, Modeller appears to function better when two or more templates are considered during homology modeling [50]. Therefore, models in this experiment were built based on different alignments resulting from multiple templates using the PSI-Coffee mode of T-Coffee. Following Perez et al. [51], the combination of selected target sequence (SmCL1) and all possible combinations of two to three selected homolog proteins resulted in 41 different alignments. For each alignment, 100 models were constructed, totalling to 4100 models for SmCL1.

Model evaluation was started from the inbuilt feature of Modeller, normalized Z-DOPE feature, a statistical method which was used to start the evaluation of 4100 models. Here a Z-DOPE score of ≤ -1 supports it to be a ‘reliable’ model. The score indicates that 80% of models’ Cα atoms are within 3.5 Å of their correct positions [52]. Only 23 models out of 4100 had score ≤ -1, hence only these were taken for further analysis (Table 3). To select the final model of SmCL1, among the 23 listed models, additional validation tools (Verify3D, PROCHECK, ProSA, ProQ, QMEAN and TM- score) were employed.

**Table 3:** Validation scores from Modeller, PROCHECK, ProQ, ProSA and Qmean for the constructed models.

Model	Alignment	Modeller	PROCHECK	ProQ	ProQ	ProSA	Qmean score	RMSD
	(PSI-coffee score)	(Z-DOPE)	(%)^a^	LG score	MaxSub	(Z-score)	(Z-score)^b^	(Å)^c^
1	X_1EWP_1M6D_1S4V_90	-1.002	89.2	4.326	0.318	-5.28	0.781	1.76
	95		10.2				(0.11)	1.77
			0					3.68
			0.5					
2	X_1EWP_1M6D_2P7U_60	-1.001	88.7	4.693	0.35	-5.4	0.767	1.79
	98		9.7				(-0.04)	1.78
			0					1.48
			1.6					
3	X_1EWP_1M6D_2XU3_78	-1.002	88.7	4.604	0.351	-5.54	0.778	1.87
	97		10.2				(0.07)	1.84
			0.5					1.77
			0.5					
4	X_1M6D_1S4V_2FO5_100	-1.065	91.9	4.808	0.38	-5.4	0.789	1.81
	95		7.5				(0.19)	3.69
			0					3.29
			0.5					
5	X_1M6D_1S4V_2FO5_93	-1.003	91.4	4.518	0.338	-5.45	0.767	1.8
	95		8.1				(-0.04)	3.74
			0					3.29
			0.5					
6	X_1M6D_1S4V_2P7U_69	-1.023	90.9	4.685	0.349	-5.37	0.796	1.78
	95		8.1				(0.27)	3.67
			0.5					1.54
			0.5					
7	X_1M6D_1S4V_2XU3_100	-1.019	88.7	4.523	0.336	-5.47	0.799	1.85
	93		10.2				(0.3)	3.69
			0					1.78
			1.1					
8	X_1M6D_1S4V_2XU3_52	-1.006	89.8	4.612	0.336	-5.39	0.746	1.85
	93		9.7				(-0.26)	3.68
			0					1.79
			0.5					
9	X_1M6D_1S4V_2XU3_53	-1.003	88.7	4.542	0.348	-5.37	0.774	1.85
	93		10.8				(0.03)	3.71
			0					1.77
			0.5					
10	X_1M6D_2FO5_2XU3_52	-1.009	88.2	4.382	0.342	-5.48	0.813	1.85
	94		10.2				(0.45)	3.29
			1.1					2.11
			0.5					
11	X_1M6D_2P7U_2XU3_34	-1.048	90.3	4.873	0.366	-5.39	0.793	1.82
	97		9.1				(0.23)	1.48
			0					1.83
			0.5					
12	X_1M6D_2P7U_2XU3_49	-1.032	90.3	4.714	0.311	-5.34	0.769	1.82
	97		9.1				(-0.02)	1.41
			0					1.81
			0.5					
13	X_1M6D_2P7U_2XU3_21	-1.012	90.9	4.764	0.349	-5.42	0.763	1.86
	97		8.6				(-0.09)	1.41
			0					1.85
			0.5					
14	X_1M6D_2P7U_2XU3_43	-1.012	89.8	4.774	0.339	-5.43	0.739	1.83
	97		9.7				(-0.33)	1.41
			0					1.83
			0.5					
15	X_1M6D_2P7U_2XU3_91	-1.012	89.8	5.101	0.366	-5.36	0.759	1.82
	97		9.1				(-0.13)	1.41
			0.5					1.88
			0.5					
16	X_1M6D_2P7U_2XU3_100	-1.012	89.8	4.596	0.326	-5.46	0.788	1.82
	97		9.1				(0.18)	1.42
			0.5					1.79
			0.5					
17	X_1M6D_2P7U_2XU3_40	-1.01	88.7	4.306	0.325	-5.41	0.769	1.78
	97		10.2				(-0.02)	1.43
			0.5					1.88
			0.5					
18	X_1M6D_2P7U_2XU3_98	-1.01	89.8	4.572	0.313	-5.36	0.763	1.82
	97		9.7				(-0.08)	1.42
			0					1.92
			0.5					
19	X_1M6D_2P7U_2XU3_72	-1.007	88.7	4.565	0.324	-5.49	0.746	1.83
	97		10.8				(-0.26)	1.41
			0					1.76
			0.5					
20	X_1M6D_2P7U_2XU3_20	-1.005	89.2	5.039	0.361	-5.42	0.78	1.83
	97		10.2				(0.09)	1.41
			0					1.79
			0.5					
21	X_1M6D_2P7U_2XU3_28	-1.005	89.8	4.525	0.311	-5.39	0.776	1.86
	97		9.7				(0.06)	1.4
			0					1.76
			0.5					
22	X_1M6D_2P7U_2XU3_25	-1.002	89.8	4.757	0.346	-5.36	0.773	1.82
	97		9.7				(0.02)	1.41
			0					1.89
			0.5					
23	X_1M6D_2XU3_37	-1.035	88.7	4.63	0.344	-5.27	0.804	1.83
	97		10.2				(0.35)	1.57
			0.5					
			0.5					

^a^Values correspond to the percentages of residues that are located in the most favourable, additionally allowed, generously allowed and disallowed regions, respectively

^b^The first value corresponds to the QMEAN global score, while the value between parenthesis refers to the QMEAN Z-score

^c^RMSD values of SmCL1 model compared to the 3D structures of the templates used for the alignment (listed in same order as column 2)

In different validation tools, there were different rules and parameters to validate the model reliability and quality. For PROCHECK analysis, a model which has 90% of amino acids of protein sequence, found in the most favourable region, is considered to be a quality model. ProQ measures the quality of the model in two ways: LG score and MaxSub. LG scores >1.5, >2.5 and >4.0 correspond to fairly well, very good and extremely good models, respectively. Similarly, MaxSub scores >0.1, >0.5 and >0.8 represent fairly well, very good and extremely good models, respectively. The Z–score of ProSA indicates the overall model quality. The value of Z-score in ProSA analysis is displayed in a plot that contains the Z-score of all the experimentally determined proteins, present in PDB. QMEAN scoring function estimates the absolute quality of modelled protein, with the models having scoring range 0 to 1 (with higher scores) are ought to be more reliable models. QMEAN Z-score used to predict the reliability of the modelled structure as compared to the high-resolution structures solved by X-ray crystallography, with an average value of 0 is considered good for a reliable model. The calculated ‘QMEAN Z-score’ provides an estimation of the degree of nativeness of the structural features observed in a model and indicates whether the model is of comparable quality to experimental structures. All the 23 models passed the quality check performed at Verify3D server [53], which derives a “3D-1D” profile based on the local environment of each residue of the protein.

Considering the above results, the model 12 (X_1M6D_2P7U_2XU3_49), listed in Table 3, was supposed to be the best model among 4100 models prepared via homology modeling using three different templates (1M6D, 2P7U and 2XU3). Model X_1M6D_2P7U_2XU3_49 with alignment score 97 has least RMSD with the templates, and was taken into consideration for further analysis.

**Fig 3:**
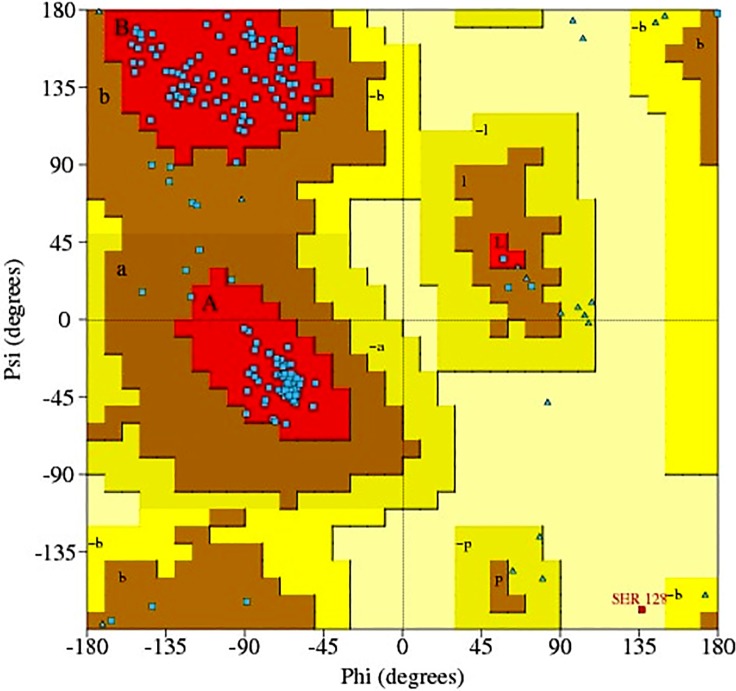
Ramachandran plot of the homology modelled structure of SmCL1. The different coloured areas indicate ‘disallowed’ (beige), ‘generously allowed’ (yellow), ‘additional allowed’ (brown), and ‘most favoured’ (red) regions.

Ramachandran plot (Fig 3), obtained from PROCHECK, was used to check the stereo-chemical quality of a given protein structure. This plot ensured the quality of the model 12 of SmCL1 with 90.3% residues in favourable region and 9.1% in additionally allowed region. Furthermore, there were only 0.5% residues in disallowed region and 0% of the residues in generously allowed region. Only Ser128 was found in the disallowed region of the plot. However, this residue can be ignored due to its far-off distance (19.4 Å) to the Cα atom of the catalytic Cys25. This suggests that Ser128 would interfere very little with the ligands binding in the active site region of SmCL1.

**Fig 4:**
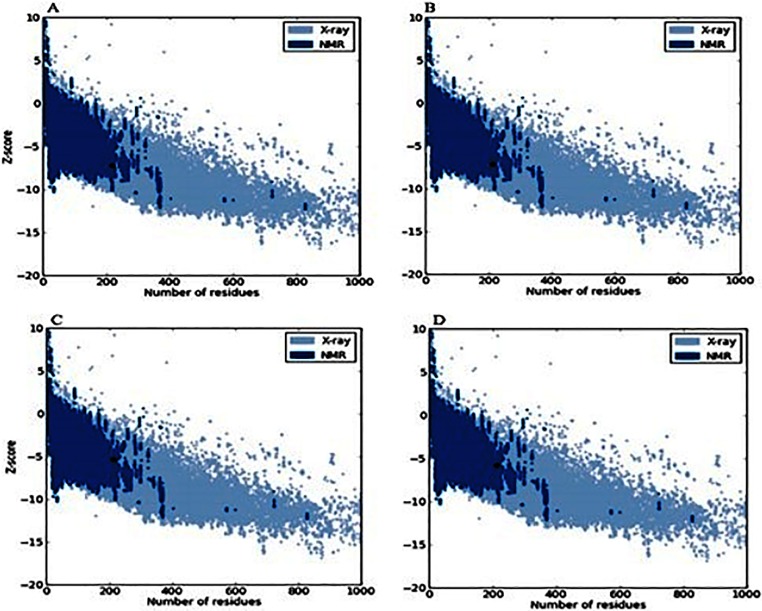
ProSA analysis for the model structure of SmCL1 (C) the template structures, 2XU3 (A), 2P7U (B) and 1M6D (D) with Z-scores values of -5.34, -7.24, -7.14 and -5.81, respectively.

**Fig 5:**
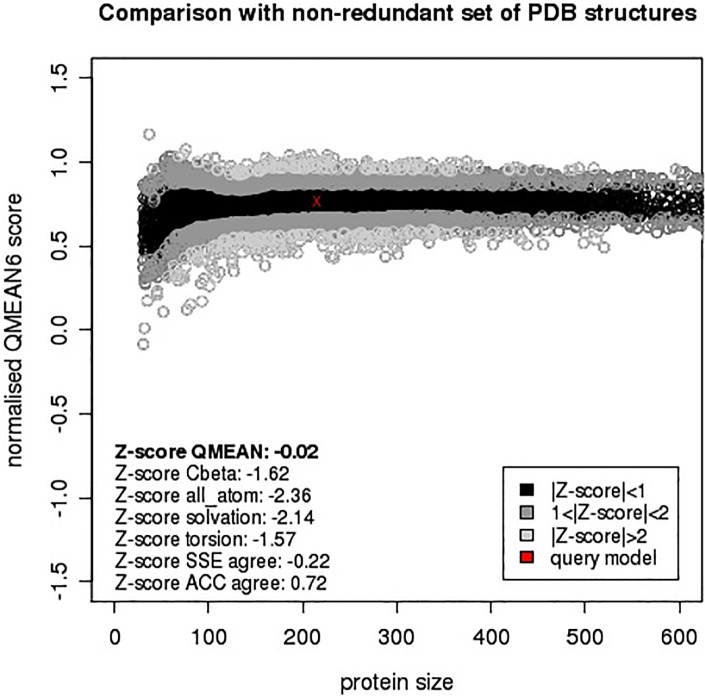
Absolute quality of SmCL1 model as assessed by QMEAN Z-score. Good models are generally located in the dark zone. The red marker indicates the positioning of SmCL1 model X_1M6D_2P7U_2XU3_49.

The ProSA analysis of the model 12 of SmCL1 was used to judge the overall quality of the protein on the basis of plot containing the Z-scores of the experimentally determined protein chains available in PDB. Fig 4 shows the Z-score plots of 2XU3 (A), 2P7U (B), model no. 12 of SmCL1 (C) and 1M6D (D), which are -7.24, -7.14, -5.34 and -5.81, respectively. Although the Z-score of model 12 is slightly lower than that of templates 2XU3 and 2P7U, it is in the same range of template 1M6D and found as a perfect fit within the structures in PDB. ProQ analysis suggests the model 12 as an extremely good model from the LG score 4.714 (i.e. >4.0), while MaxSub score 0.311 (i.e. >0.1) ranks it to be a fairly well model. QMEAN score of this model, 0.769 (Z-score -0.02), localized in dark zone, confirms the model X_1M6D_2P7U_2XU3_49 as a reliable one (Fig 5).

**Fig 6:**
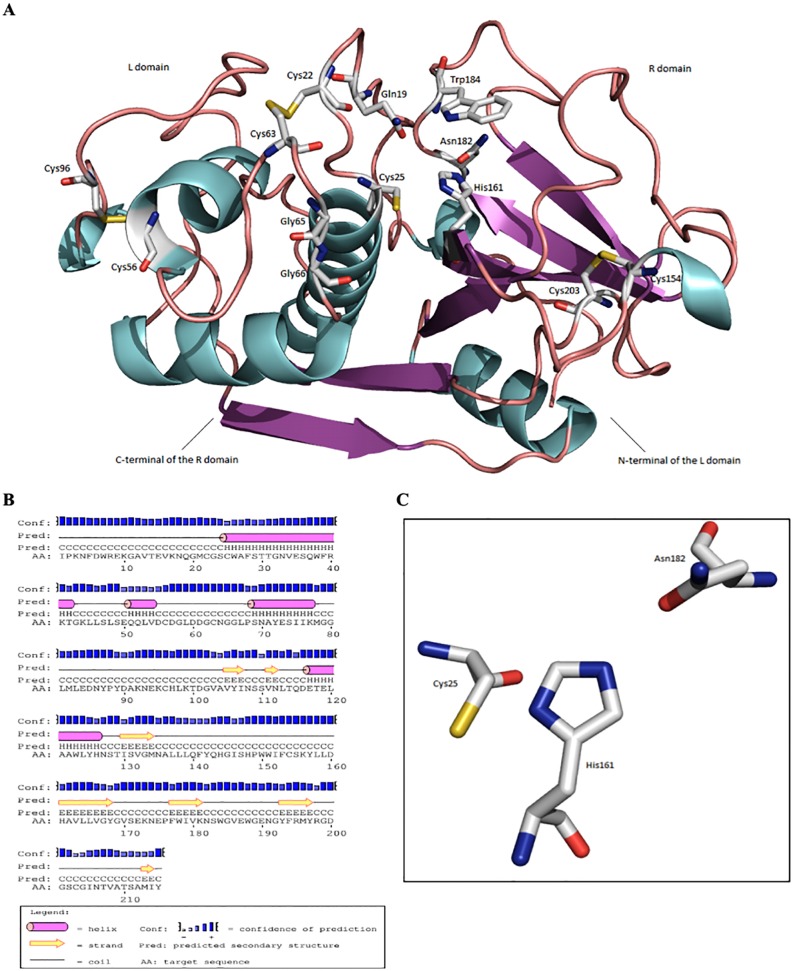
Representation of modelled structure, secondary structure and active site residues of SmCL1. (A) Cartoon view of modelled structure of SmCL1, where some conserved catalytic residues and disulphide bonds are represented. The model contains two typical L (N-terminal) and R (C-terminal) domains of papain-like cysteine proteases sharing ‘‘V” shaped active site. (B) Predicted secondary structure of SmCL1 using PSIPRED server. (C) The active site residues (Cys25, His161, Asn182) of SmCL1.

SmCL1 is systematized into cysteine protease [EC.3.4.22.XX] group belonging to papain-superfamily CA, family C1A and utilizes important catalytic residues like glutamine (Gln19), cysteine (Cys25), histidine (His161) and asparagine (Asn182). Residues that constitute the different binding pockets remain strictly conserved in three catalytic regions (shown in the boxes) as presented in Fig 2. Modelled SmCL1 represents highly conserved framework structure of papain-like cysteine protease composed of two domains. The first one is largely α-helix-rich (L) domain and the second one is predominantly β-sheet-rich (R) domain, separated by a groove containing the active site (Fig 6A). There are four α-helices in the L domain of the modelled SmCL1 and six β-sheets in the R domain. Apart from these, three small α-helices are also seen at the surface of the model, which resembles the typical features of C1 papain-like fold [54].

To stabilize the binding region, the C-terminal of R domain and N-terminal of L domain bind to the L and R domains, respectively. SmCL1 model includes seven cysteine residues, which are common to papain family (Fig 6A). Among these, six cysteine residues (Cys22-Cys63, Cys56-Cys96 and Cys154-Cys203) are involved in the disulphide bonds. The seventh one, Cys25, corresponds to the active catalytic residue. Cys25 together with His161 and Asn182 form the catalytic triad (Fig 6C). Gln19 along with Trp184 is another important and conserved moiety of this family, whose side chain forms the ‘oxyanion hole’; this is considered as an important feature of the enzymes with proteolytic activity. Residues Gly65 and Gly66 correspond to conserved glycine rich region, which is a typical feature of this family, ensuring the stability of the complex by forming number of hydrogen bonds with the substrate [55]. SmCL1 also contains the tripeptide consensus sequence Arg-Gly-Asp (RGD) binding motif which aids in cellular targeting (Fig 2). It serves to anchor proteins that have no trans-membrane spanning domain or do not possess a GPI moiety. The secondary structure of SmCL1 as predicted from PSIPRED server [56] supports the three dimensional structure of the protein (Fig 6B).

**Fig 7:**
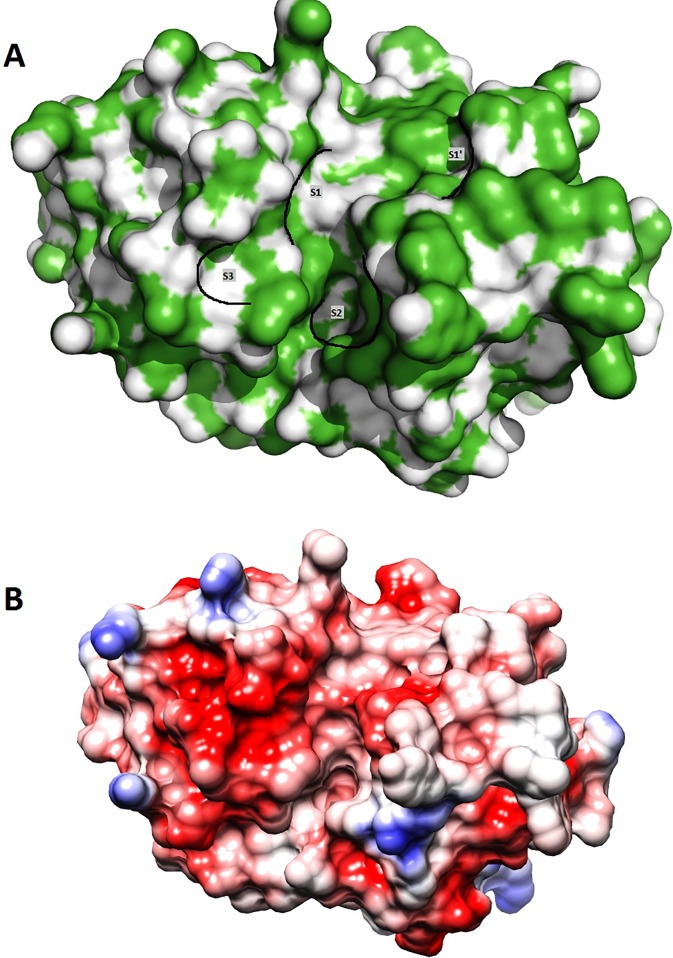
Binding pockets and electrostatic potential of modelled SmCL1. (A) Surface view of the modelled SmCL1, with marked S1', S1, S2 and S3 pockets. (B) Surface view of the modelled SmCL1 illustrating the electrostatic potential, where red corresponds to negative potential and blue to positive potential.

The active site of papain-like protease SmCL1 is usually lined by four pockets: S1, S1', S2 and S3 [57] (Fig 7A). Among these, S1 and S2 are the least and most defined pockets, respectively. S2 pocket residues bestow major specificity to the P2 substrate residues. The major residues lining the four distinct binding pockets of cathepsin SmCL1 of *S*. *mansoni* are listed in Table 4.

**Table 4:** Important residues lining the binding pockets of SmCL1 of *Schistosoma mansoni* and cruzain of *Trypanosoma cruzi*.

Cysteine protease	S1'	S1	S2	S3
SmCL1	Ala136, **Leu137**, **Gln140**, Asp160, Trp184	Gly23, Ser24, **Asn64**, Gly65, Gly66	Leu67, **Pro68, Gly133,** Leu159, **Ala162, Val208**	Asp60, **Asp61**
Cruzain	Ala141, **Ser142**, **Met145**, Asp161, Trp184	Gly23, Ser24, **Ser64**, Gly65, Gly66	Leu67, **Met68, Ala138,** Leu160, **Gly163, Glu208**	Asp60, **Ser61**

Phylogenetic analysis clearly indicates that SmCL1 is related to cruzain from the parasitic protozoan *T*. *cruzi*, supporting that SmCL1 and cruzain are the members of a separate clade of C1 peptidases, termed as “cruzain lineage” [58,59]. To supplement the results of phylogenetic analysis, the protein sequences of SmCL1 and cruzain (PDB ID: 1EWP_A) are compared in Fig 2. It is found that the enzymes have not diverged at the catalytic triad residues and at the “ERFNAAQ/A” motif in the pro-region peptide. Analysis of the deduced primary structure of SmCL1 also revealed that it shares 45% sequence identity with cruzain (Table 2). Moreover, very little differences are found in the residues lining the binding pockets of SmCL1 and cruzain (Table 4). Residues highlighted in Table 4 are the ones which are not present in similar order. Other residues like Ala, Asp, Trp in S1'; Gly, Ser in S1; Leu in S2; Asp in S3 are present in similar order and lining these pockets. The electrostatic potential of the modelled SmCL1 is shown in Fig 7B, where the red surface colour corresponds to negative potential and the blue one corresponds to positive potential.

**Fig 8:**
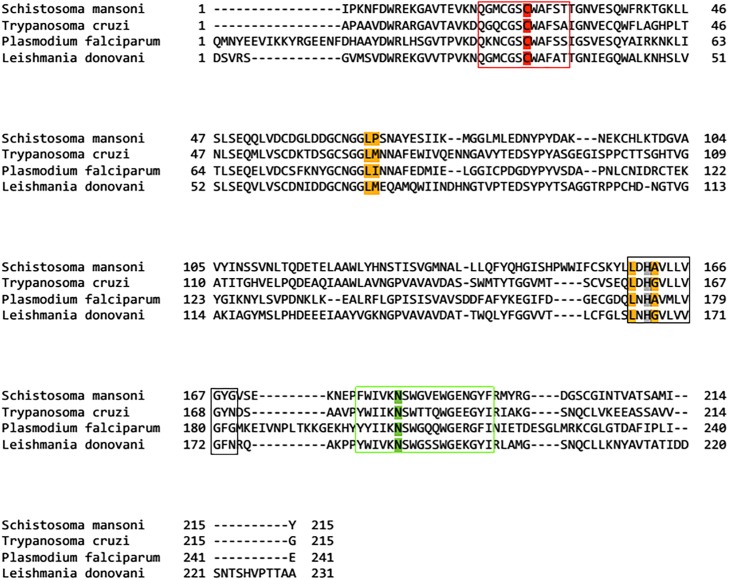
Multiple sequence alignment of SmCL1 of *Schistosoma mansoni* and cysteine protease of related protozoan parasites. Active site residues (Cys 25, His 161 and Asn182) are found conserved in SmCL1 and similar enzyme of different parasites. The red, black and green boxes show the conserved region alongside the active sites Cys25 (red), His161 (grey) and Asn182 (green), respectively. Important S2 pocket residues found conserved are marked yellow.

The availability of the theoretical 3D model of SmCL1 using homology modeling protocol leads to the docking procedure. As discussed earlier, this experiment aims to justify a couple of important facts; one among them is the importance of non-peptide inhibitors over the peptide ones and the other being the ‘better’ activity of phytochemicals against non-phytochemical inhibitors. Since, no elaborated studies have been reported on phytochemicals against *S*. *mansoni*, during library preparation all the phytochemicals which showed anti-parasitic property against *T*. *cruzi*, *P*. *falciparum* and *L*. *donovani* were considered. This consideration seems to be justified, as SmCL1 is closely related to the cysteine protease of the above mentioned parasites on the basis of the important conserved catalytic triad and S2 binding pocket residues (Fig 8). Keeping in view these facts, two different libraries were prepared; one had non-peptide non-phytochemicals inhibitors and the other included non-peptide phytochemicals.

Docking was performed for all the molecules in the two libraries. After docking, only few drug-like compounds were considered as ‘best compounds/ligands’ against SmCL1 on the basis of the following observations using PyMOL: (1) Adopted position and orientation of the ligand in the SmCL1 binding site, (2) Ligand covering/interacting with active site residues, (3) Ligand interacting with active site and S2 pocket residues and (4) Ligand interacting with S1 pocket residues.

The results of ‘14 best docks/leads’ from ligand library comprising 60 orally active drug-like compounds from ZINC Database are listed in Table 5 and the results of ‘13 best docks/leads’ from ligand library of 55 orally active drug-like phytochemicals are listed in Table 6. Tables 5 and 6 includes the ZINC ID/PubChem ID, ligand name, binding energy (Kcal/mol) and inhibition constant (μM) of the ligands in the two libraries docked with the target protein SmCL1. S1 and S2 Tables include the ZINC ID/PubChem ID and the structure of the best leads included in the two libraries.

**Table 5:** Best docks from the ligand library prepared from ZINC Database.

Ligand/ZINC ID	Ligand name	Binding Energy (Kcal/Mol)	Inhibition Constant (μM)
ZINC_1225898	N'~2~,N'~6~-bis(2-hydroxybenzylidene)-2,6-pyridinedicarbohydrazide	-5.59	80.15
ZINC_43071156	5-(2-aminophenyl)-N-[5-(4-chlorophenyl)-1,3,4-thiadiazol-2-yl]-1,3,4-oxadiazole-2-carboxamide	-5.79	57.39
ZINC_8691187	(5-chloro-2-methoxy- phenyl) carbamoyl methyl	-4.93	242.86
ZINC_20191037	8-chloro-N-[3-(morpholin-4-yl)propyl]-5H-pyrimido[5,4-b]indol-4-amine	-4.66	387.05
ZINC_6094108	5-iodo-3-sulfanylcarbonimidoylazo-1H-indol-2-ol	-4.38	613.58
ZINC_46442	2-methoxy-1-naphthaldehyde Thiosemicarbazone	-4.21	816.45
ZINC_17953024	(3Z)-N,5-bis(4-chlorophenyl)-3-[(1-methylethyl)imino]-3,5-dihydrophenazin-2-amine	-6.66	13.18
ZINC_344779	(2E)-2-{[5-(3-chlorophenyl)furan-2-yl]methylidene}hydrazinecarbothioamide	-5.13	173.25
ZINC_22001688	N-{4-Oxo-2-(1H-tetrazol-5-yl)-4H-1-benzopyran-8-yl]-4-(4-phenylbutoxy) benzamide	-5.09	184.54
ZINC_13474224	(4R)-3-(3-bromophenyl)-4-methyl-4,5-dihydropyrazole-1-carbothioamide	-4.57	450.32
ZINC_2207043	Ethyl-5-cyano-4-(furan-2-yl)-6-((2-oxo-2-(p-tolylamino)ethyl)thio)-2-phenyl-1,4-dihydropyridine-3-carboxylate	-4.24	777.98
ZINC_1228042	4,4'-[(2-chloro-1,4-phenylene) bis(nitrilomethylylidene)] di(1,3-benzenediol)	-4.71	354.17
ZINC_12403599	1-(4-biphenylyl) ethanonethiosemicarbazone	-5.55	85.0
ZINC_13580132	(E)-2-(1-(4-phenoxyphenyl) ethylidene)hydrazine carbothioamide	-5.36	118.21

**Table 6:** Best docks from the ligand library from Phytochemical Database.

Ligand/PubChem ID	Ligand name	Binding energy (Kcal/mol)	Inhibition constant (μM)
CID 5458457	Ailanthinone	-7.81	1.89
CID 91503	Dihydroquinidine	-7.37	3.98
CID 92766	Corynanthine	-7.33	4.24
CID 44576034	Akuammine	-7.01	7.22
CID 6711208	Simalikalactone-D	-7.01	7.26
CID 9851692	Beta-dichroine	-6.79	10.54
CID 173866	Ajugarin-1	-6.67	12.99
CID 5318998	Licochalcone A	-6.56	15.63
CID 5280961	Genistein	-5.99	40.73
CID 73255	Voacangine	-5.89	48.3
CID 162464	Cirsilineol	-5.46	98.91
CID 12304613	Thujone	-5.07	193.54
CID 97214	Eupatorine	-4.97	225.73

**Fig 9:**
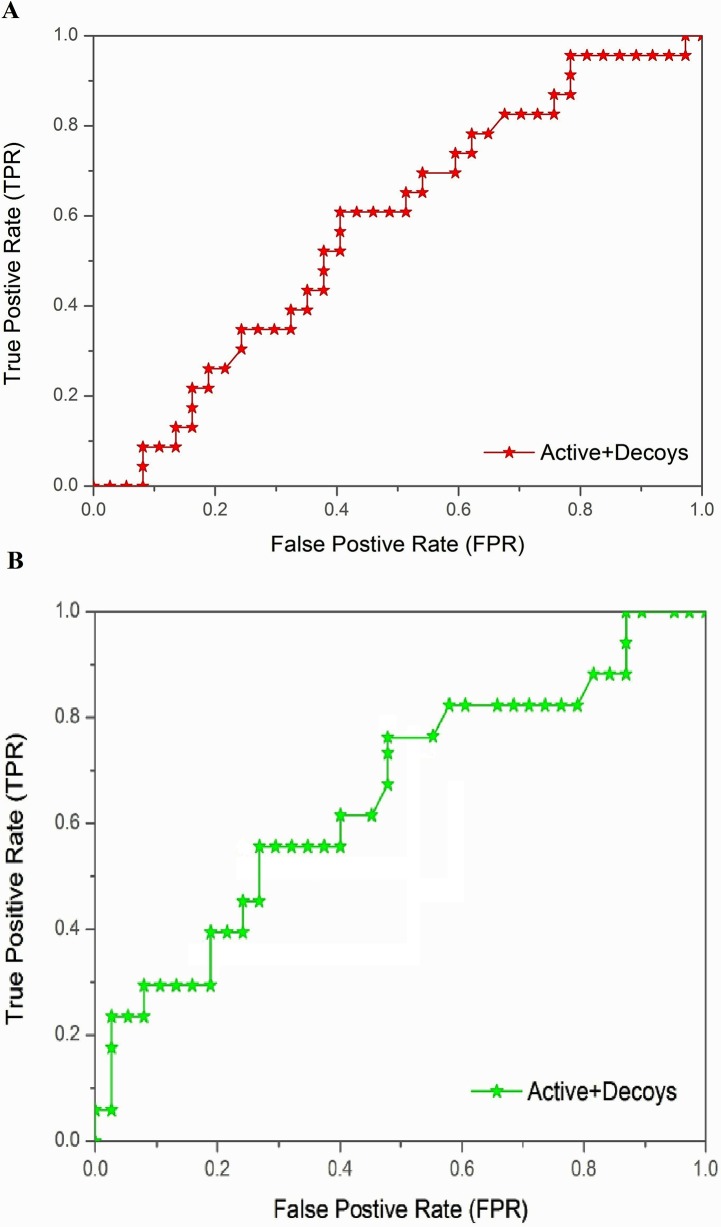
Validation of the docking protocol via ROC analysis. (A) ROC curve for ZINC leads (score 0.56). (B) ROC curve for phytochemical leads (score 0.67).

The docking results were evaluated by ROC curves, which describe the trade-off between “sensitivity” and “specificity”. “Sensitivity” is the ability of the classifier to detect true positives, while “specificity” is the ability to avoid false positives. The area under an ROC curve indicates the quality of enrichment. The ROC value of a random classifier is 0.5, while that of an excellent classifier is greater than 0.9 [60]. ROC curve analysis was performed using the ‘binding energy’ results of docked ligands to find out the relation between the true positives and true negatives from two different libraries. Actives and decoys were labelled 1 and 0, respectively; the actives were those ligands which were found interacting with important residues (active site and S2 pocket) of SmCL1 and otherwise were supposed to be decoys. The ROC score of the ZINC leads was 0.56, whereas the same of phytochemicals was 0.67. Thus, ROC curve analysis (Fig 9A for ZINC compounds and Fig 9B for phytochemicals) suggests that library of phytochemicals has larger number of the actives than decoys, supporting the effectiveness of the phytochemicals as potential leads against SmCL1.

For thorough analysis of the activities of leads and to support that the phytochemicals could be better and potential drug against SmCL1, one best lead was picked from each library. Using PyMOL, the inter-molecular interactions of the docked ligands with SmCL1 were observed and the bond distance (Å) was measured between the interacting residues to finalize the best lead from each library. Through careful observations, compound with ZINC ID 22001688 and phytochemical *Simalikalactone-D* (PubChem ID: CID 6711208) were found as the best lead compounds from their respective libraries.

**Fig 10:**
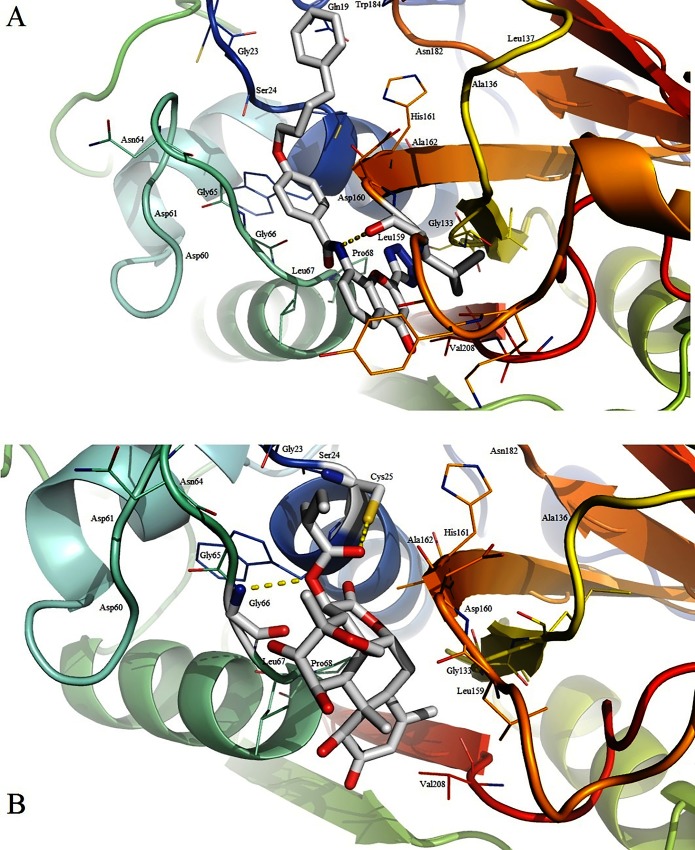
Binding modes of ZINC lead 22001688 (A) and *Simalikalactone-D* (B) into SmCL1 binding site.

**Fig 11:**
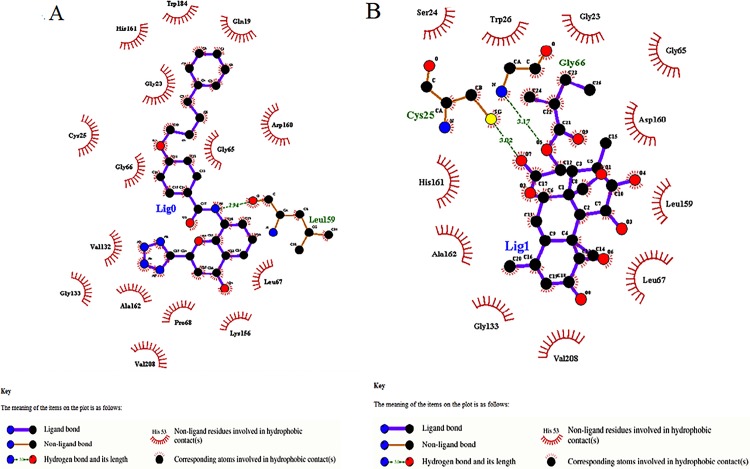
Detailed interaction view of ZINC lead 22001688 (A) and *Simalikalactone-D* (B) with SmCL1 generated by Ligplot.

**Fig 12:**
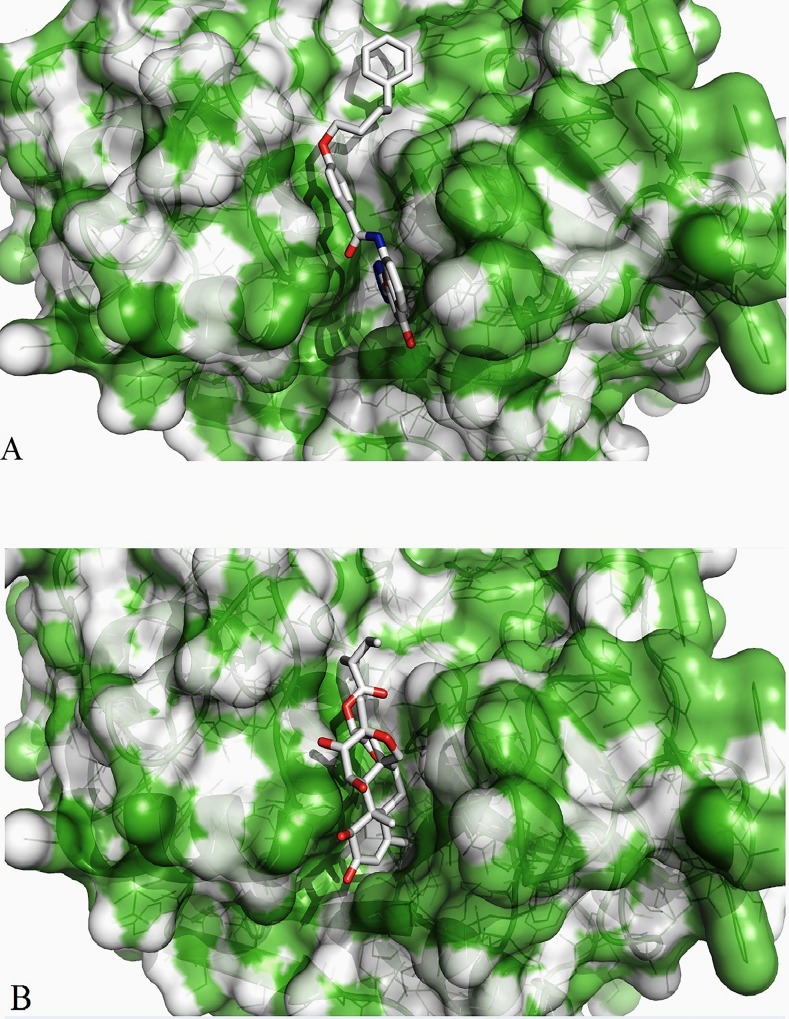
Surface view of interaction of ZINC lead 22001688 (A) and *Simalikalactone-D* (B) with SmCL1.

ZINC lead 22001688 was found interacting with Leu159 (S2 pocket residue) of SmCL1 through the hydrogen bond of length 2.94 Å (Figs 10A and 11A). *Simalikalactone-D* was found interacting with two important residues, Cys25 (active site residue) and Gly66 (S1 pocket residue) with hydrogen bond of lengths 3.02 Å and 3.17 Å, respectively (Figs 10B and 11B). Interactions of these lead compounds with SmCL1 were analysed using PyMol (Fig 10) and Ligplot (Fig 11). Plot illustrating active site interactions (hydrogen bond interaction with Cys25 and hydrophobic interaction with His161) of *Simalikalactone-D* (Fig 11B) suggests it to be a better lead as compared to ZINC lead 22001688 (Fig 11A). Further, the surface view for the lead interactions with SmCL1 (Fig 12 A and 12B) confirms that *Simalikalactone-D* covers the active site and fits best in the pockets of SmCL1 (Fig 12B), and hence it could be an effective inhibitor against SmCL1.

**Fig 13:**
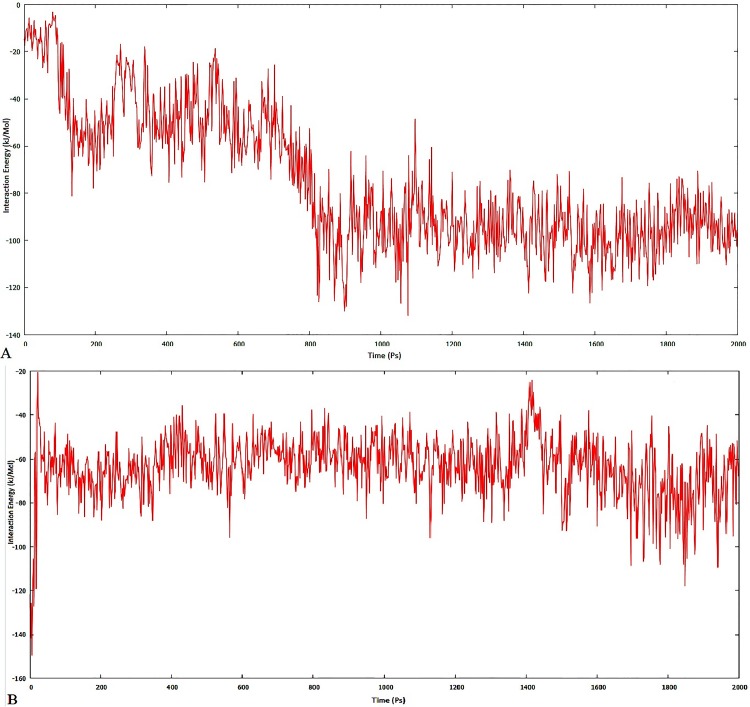
Interaction energy plot for 2 ns MD simulation of SmCL1 with ZINC lead 22001688 (A) and *Simalikalactone-D* (B).

**Fig 14:**
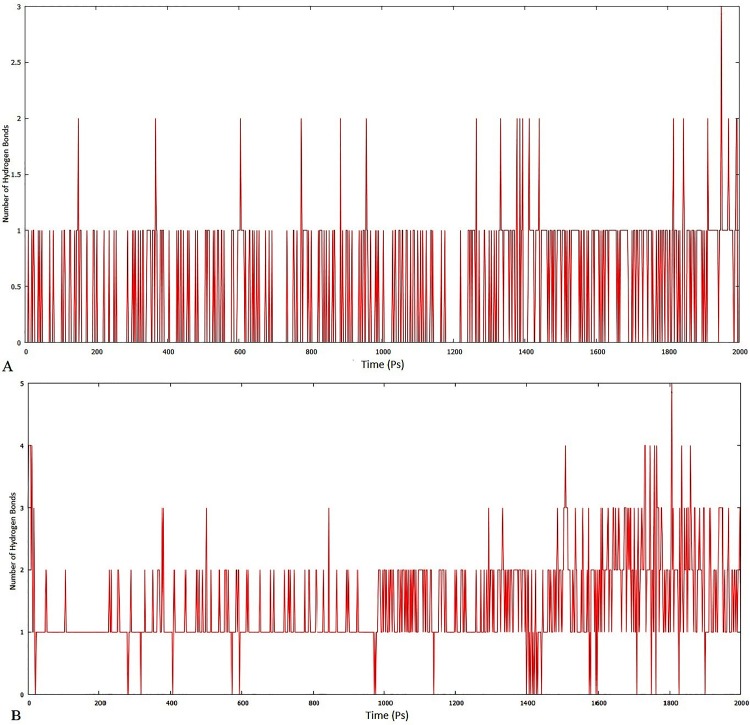
Stability evaluation of docked complexes of ZINC lead 22001688 (A) and *Simalikalactone-D* (B) using hydrogen bonding pattern as a function of time.

To confirm the stability of each lead compound, MD simulation was performed at 2000ps. The results were observed on the basis of plots obtained for interaction energy and hydrogen bond variation at various time intervals. Negative plot for interaction energy reflects the association of the complex and positive plot suggests the dissociation of the same complex. The interaction energy plot of ZINC lead 22001688 (Fig 13A) suggests it to be non-stable interaction, where the association and dissociation takes place until 1100ps, which later stabilizes reaching the time interval of 1200ps. In the case of *Simalikalactone-D* (Fig 13B), the variation in interaction energy was found to be insignificant and was more stable from 600ps to 1400ps. Further, the hydrogen bond interactions of ZINC lead 22001688 reaches a maximum of three and remains one for most of the time (Fig 14A); whereas, in the case of *Simalikalactone-D* (Fig 14B), the hydrogen interaction number reaches five and for most of the time remains two. This illustration of greater hydrogen interaction number further confirms the greater stability of the phytochemical *Simalikalactone-D*.

**Fig 15:**
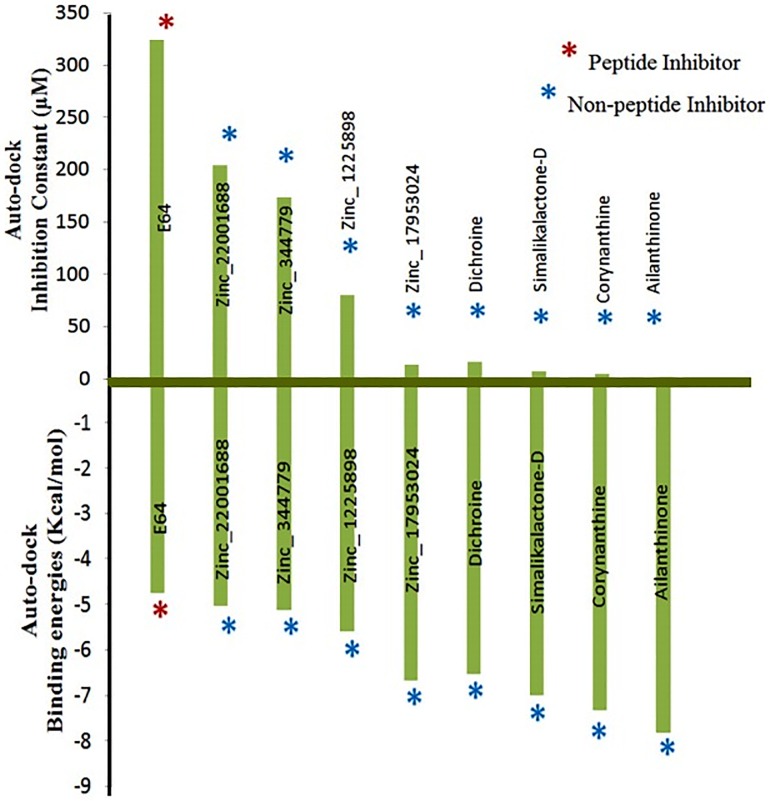
Inhibition constant and binding energies of reference and screened compounds. The reference compound is E64 (peptide inhibitor) while rest are screened non-peptide compounds.

In support of this experiment, when complete data was analyzed, the activity of non-peptide non-phytochemical and phytochemical inhibitors against cathepsin SmCL1 could be best illustrated by the bar graph (Fig 15). Non-peptide inhibitors, ZINC lead 22001688 and *Simalikalactone-D*, were found to show good results both for inhibition constant and interaction energies in comparison to E64, which is a reported peptide-based inhibitor against SmCL1. Further, if we perform comparative analysis of AutoDock results for four good leads from each of the two libraries, the results show that the phytochemical inhibitors have lower inhibition constant and more negative of interaction energy than non-phytochemical inhibitors, which makes it quite clear that phytochemicals like *Simalikalactone-D* could be the better drug against the control of schistosomiasis.

Since the actual synthesis and testing of above screened inhibitors is beyond the resources available to us, an attempt was made to validate our computational hypothesis by linking it to reported experimental work. As mentioned earlier, peptide inhibitors such as peptidyl diazomethyl ketones [9], fluoromethyl ketones (FMK) [15] and vinyl sulphones (VS) [17] have shown to inhibit the activity of SmCL1. In order to compare the experimental activity of reported peptide inhibitors [9, 15, 17] with SmCL1 model, molecular docking approach was implemented. As evident from the AutoDock results (S3 Table), the peptide inhibitors exhibit less negative interaction energy (-3.14 to -4.91 Kcal/mol), high inhibition constant (251.93 μM—4.77 mM), and follow the similar trend of increase/decrease in Ki values as reported in the experimental work [9, 15]. It has been reported that Mu-F-nitroR-FMK is the only FMK inhibitor that has the least effect on haemoglobin degradation [15]; this result has also been verified by docking protocol giving rise to mM (higher) value of inhibition constant (S3 Table). Cysteine protease inhibitors (CPIs) against *S*. *mansoni* infection have shown to prevent growth and differentiation of the parasite. Among the CPIs, the best schistosomicidal effect was observed with FMK followed by VS to selectively arrest parasite replication [61]. Using docking protocol (S3 Table), we have shown that FMK (Mu-Y-(O-methyl)-hF-FMK) exhibit more negative interaction energy and lower inhibition constant as compared to K11777 (VS), suggesting FMK as better peptide inhibitors against SmCL1.

For thorough analysis of activities of FMK (Mu-Y-(O-methyl)-hF-FMK) and VS (K11777), MD simulation was performed at 2ns. The interaction energy plot of Mu-Y-(O-methyl)-hF-FMK (S1A Fig) suggests it to be non-stable interaction, where association and dissociation takes place until 750 ps, which later stabilizes reaching time interval of 1000 ps. In case of K11777 (S1C Fig), the variation in interaction energy was much higher suggesting it to be more non-stable interaction as compared to Mu-Y-(O-methyl)-hF-FMK; the association and dissociation in K11777 takes place until 750 ps, which later destabilises and dissociation continues till the end of MD run. Further, in the case of Mu-Y-(O-methyl)-hF-FMK, the hydrogen interaction number (S1B Fig) reaches two and for most of time remains one in number; whereas, in the case of K11777, the hydrogen interaction number (S1D Fig) reaches two from 250–750 ps and for much lesser time remains one in number. This illustration further confirms the greater stability of Mu-Y-(O-methyl)-hF-FMK over K11777.

The unavailability of experimental information on non-peptide inhibitors against SmCL1 made it necessary to identify and select the reported non-peptide inhibitors (plant and non-plant) of cysteine protease closely related to SmCL1. As mentioned earlier, cruzain from the parasite *T*. *cruzi* is closely related to SmCL1; the two enzymes have not diverged at the catalytic triad and the binding pocket residues are also found conserved (Table 4). S2 Fig clearly shows the superimposed image of SmCL1 (cyan colour) on cruzain (grey colour) highlighting the conserved residues of active site and similarly conserved residues lining S1’, S1, S2 and S3 pockets. Considering the above fact, cruzain non-peptide inhibitors were tested against SmCL1 via docking protocol to estimate the interaction energy and inhibition constant parameters. The parameters obtained were used to analyse the inhibition pattern by comparing it with the reported cruzain inhibition values [62–65].

As evident from the AutoDock results (S4 Table), non-peptide phytochemical inhibitors exhibit more negative interaction energy (> -7.00 Kcal/mol) and lower inhibition constant (< 10μM) against SmCL1 as in case of cruzain with lower reported IC50 values [62]. Further, AutoDock results for non-peptide non-phytochemical inhibitors (S4 Table) showed that Nequimed, thiazolidinones and thiosemicarbazone inhibitors exhibit less negative interaction energy (< -7.00 Kcal/mol) and higher inhibition constant (> 10μM) against SmCL1, which is in consistence with the reported cruzain inhibition parameters [63–65]. It was also found that non-peptide non-phytochemical and phytochemical inhibitors show more negative interaction energy and lower inhibition constant as compared to the values of peptide inhibitors against SmCL1. Further, it was interesting to observe that cruzain inactive non-peptide inhibitors act as decoys for SmCL1 too, as shown by the mM values of AutoDock inhibition constant (S4 Table).

To analyse the stability of cruzain non-peptide inhibitors against SmCL1, MD simulation was performed at 2 ns. The interaction energy plot (S3A Fig) of dihydrochalcone-compound 3 (best hit due to interaction with active site residue) suggests it to be a stable complex with lesser energy variation over the MD run. The hydrogen interactions (S3B Fig) for compound 3 reach a maximum of five and remain 2 or 3 for most of the time. Out of the non-peptide non-phytochemical inhibitors, Neq 42 and thiazolidinones (4p) energy plots (S3C and S3G Fig) suggest them as slight non-stable complexes, where the association takes place till 1750 ps, which later destabilises resulting in dissociation of the complex. The hydrogen interactions (S3D Fig) for Neq 42 reach a maximum of three and remain one for most of the time, whereas for thiazolidinones (4p) (S3H Fig) alternate between one and two over 2 ns. As discussed above, docking protocol identified that cruzain inactive non-peptide inhibitors (Neq 175 and thiazolodinones-4k) act as decoys for SmCL1 too; the same was also proved by MD simulation. The interaction energy (S3E and S3I Fig) and hydrogen interaction (S3F and S3J Fig) plots suggest that Neq 175 and thiazolidinones (4k) exhibit low energy values (-20 Kcal/mol) and null hydrogen interactions over 2 ns.

**Fig 16:**
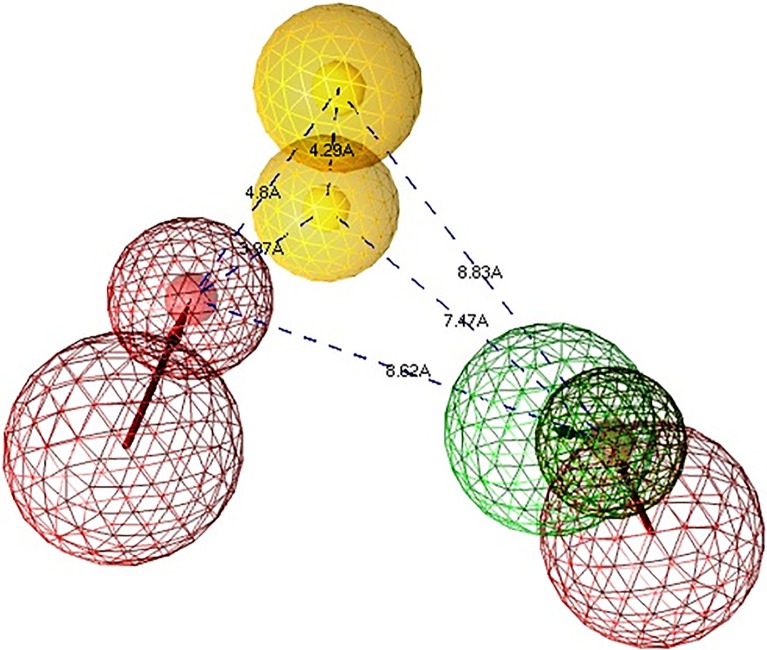
Pharmacophore features of *Simalikalactone-D* represented via Ligand Scout. The coloured spheres represent the pharmacophore features of the lead: yellow sphere for hydrophobic interaction; red and green spheres for hydrogen bond acceptor and donor, respectively.

**Fig 17:**
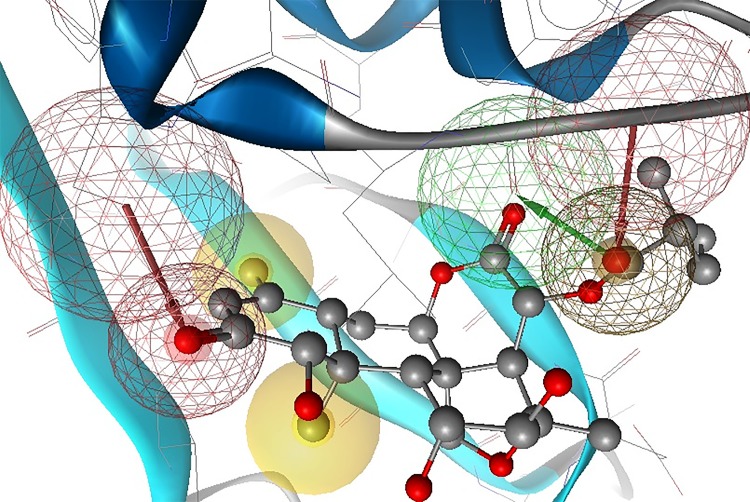
Typical Pharmacophore features of *Simalikalactone-D* interacting with SmCL1 via Ligand Scout.

In view of the above results and discussion, we may suggest that phytochemicals are the better leads. Therefore, *Simalikalactone-D* could be a good fit for the pharmacophore model generation and validation. Pharmacophore features of SmCL1-*Simalikalactone-D* complex were identified using Ligand Scout v3.12 as shown in Figs 16 and 17. Based on the interaction points between the ligand and protein SmCL1, two hydrogen bond acceptor (HA), one hydrogen bond donor (HD) and two hydrophobic (HY) chemical features were identified in classifying the pharmacophore model resulting from SmCL1-*Simalikalactone-D* docked complex. Inter-feature distance constraints (Fig 16) were calculated between different pharmacophoric features. The given inter-feature distances between HA and HA/HD, HA and HY, HY and HY, HY and HA/HD could be used to map new lead molecules whose chemical groups should be easily fitted in the 3D triangle formed of the given dimensions (Fig 17). Using pharmacophore features (Figs 16 and 17) of *Simalikalactone-D*, one can devise wise set of rules to screen more potential inhibitors against SmCL1, thus preventing the neglected tropical disease ‘schistosomiasis’. Further investigations are in progress in our laboratory to validate the pharmacophore features of *Simalikalactone-D* and identify new and potential hits for future drug design.

## Conclusions

An effort has been made to propose some probable leads against the SmCL1 whose pharmacophore features may be considered for further investigation in devising efficient drugs to retard the hazardous proliferation of *S*. *mansoni*. According to the available literature, SmCL1 could be the potential drug target in *S*. *mansoni*. The unavailability of 3D crystal structure of SmCL1 in PDB necessitated predicting the best possible theoretical model of this protein via homology modeling protocol. Virtual screening of potential drugs was carried out using the Autodock suite. The best possible library of phytochemicals and their leads were confirmed through ROC analysis and MD simulations. The scope of this investigation is to support cost-effective experimental screening of reported drugs against SmCL1. In addition to the efficacy of the proposed non-peptide leads, our work also suggested that phytochemicals could be better inhibitors against *S*. *mansoni*. Pharmacophore features of the proposed lead *Simalikalactone-D* should be given considerable importance and need to be studied for future screening of related lead compounds for developing noble drugs against SmCL1, thereby putting a check on neglected tropical disease ‘schistosomiasis’.

## Supporting Information

S1 Fig Interaction energy and number of hydrogen bonding plot of peptide inhibitor-SmCL1 complex during 2 ns MD simulation. **(**A) and (B) represent the interaction energy and number of hydrogen bond of Mu-Y-(O-methyl)-hF-FMK, respectively; whereas, (C) and (D) represent interaction energy and number of hydrogen bond of K11777, respectively, against SmCL1 during 2 ns simulation.
(TIF)

S2 Fig SmCL1 model (cyan colour) superimposed over cruzain model (grey colour). The active site residues marked with * and highlighted with pink colour, forms catalytic triad and remains conserved. The other important residues are also found conserved, i.e. Leu67, Leu159 of S2 pocket (highlighted blue); Gly23, Gly65, Gly66 of S1 pocket (highlighted green); Asp60 of S3 pocket (highlighted yellow); Trp184 of S1’ pocket (highlighted brown).
(TIF)

S3 Fig Interaction energy and number of hydrogen bonding plot of non-peptide inhibitor-SmCL1 complex during 2 ns MD simulation. (A), (C), (E), (G) and (I) represent the interaction energy of Compound 3 (Dihydrochalcones), Neq42, Neq175,4p (Thiazolidinones) and 4k (Thiazolidinones), respectively; whereas, (B), (D), (F), (H) and (J) represent the number of hydrogen bonds of the Compound 3 (Dihydrochalcones), Neq42, Neq175, 4p (Thiazolidinones) and 4k (Thiazolidinones), respectively, against SmCL1 during 2 ns simulation.
(TIF)

S1 Table Ligand structure of best docks from ligand library prepared from ZINC Database. (PDF)

S2 Table Ligand structure of best docks from ligand library prepared from Phytochemical Database. (PDF)

S3 Table AutoDock results (binding energy and inhibition constant) and reported inhibition constant (Ki) of peptide based inhibitors against SmCL1. (PDF)

S4 Table AutoDock results (binding energy and inhibition constant) of non-peptide cruzain inhibitors docked with SmCL1 and reported inhibition parameters of cruzain. (PDF)
