# Supplementary figures and images for: Novel Non-Peptide Inhibitors against SmCL1 of Schistosoma mansoni: In Silico Elucidation, Implications and Evaluation via Knowledge Based Drug Discovery
Source: PLoS One. 2015 May 1;10(5):e0123996. doi: 10.1371/journal.pone.0123996 (PMC4416924; doi:10.1371/journal.pone.0123996)

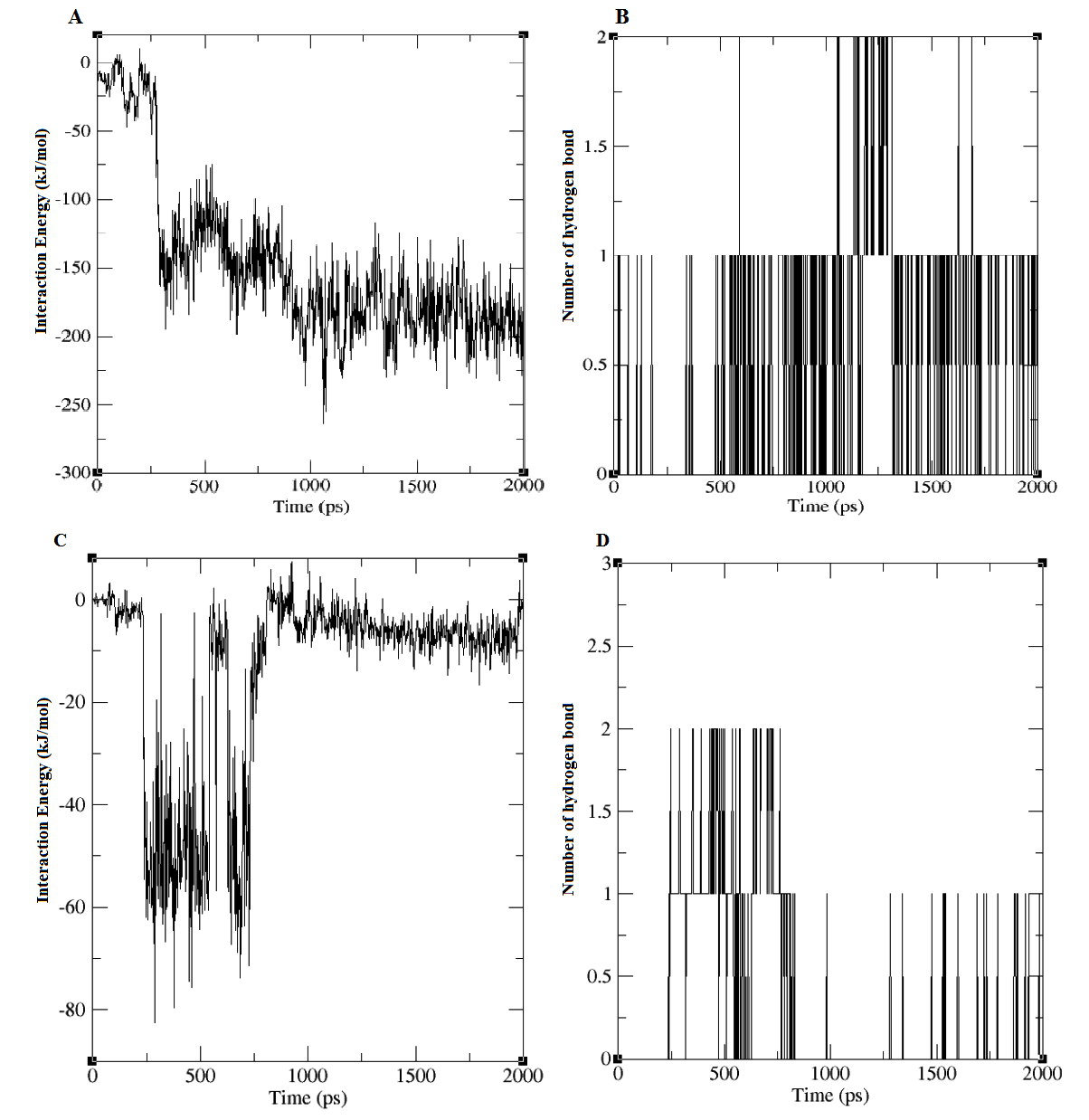

Supplement: S1 Fig — (A) and (B) represent the interaction energy and number of hydrogen bond of Mu-Y-(O-methyl)-hF-FMK, respectively; whereas, (C) and (D) represent interaction energy and number of hydrogen bond of K11777, respectively, against SmCL1 during 2 ns simulation. (TIF) [file pone.0123996.s001.tif]

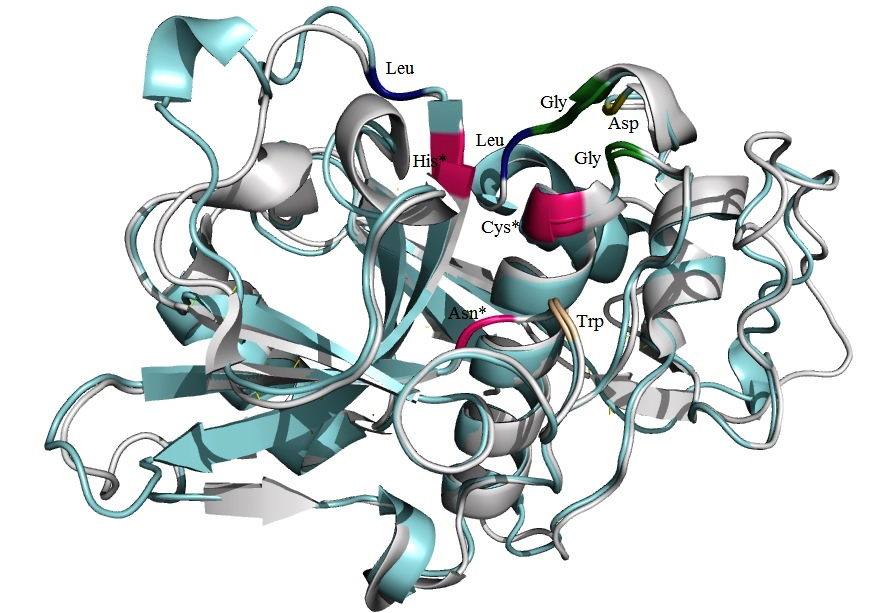

Supplement: S2 Fig — The active site residues marked with * and highlighted with pink colour, forms catalytic triad and remains conserved. The other important residues are also found conserved, i.e. Leu67, Leu159 of S2 pocket (highlighted blue); Gly23, Gly65, Gly66 of S1 pocket (highlighted green); Asp60 of S3 pocket (highlighted yellow); Trp184 of S1’ pocket (highlighted brown). (TIF) [file pone.0123996.s002.tif]

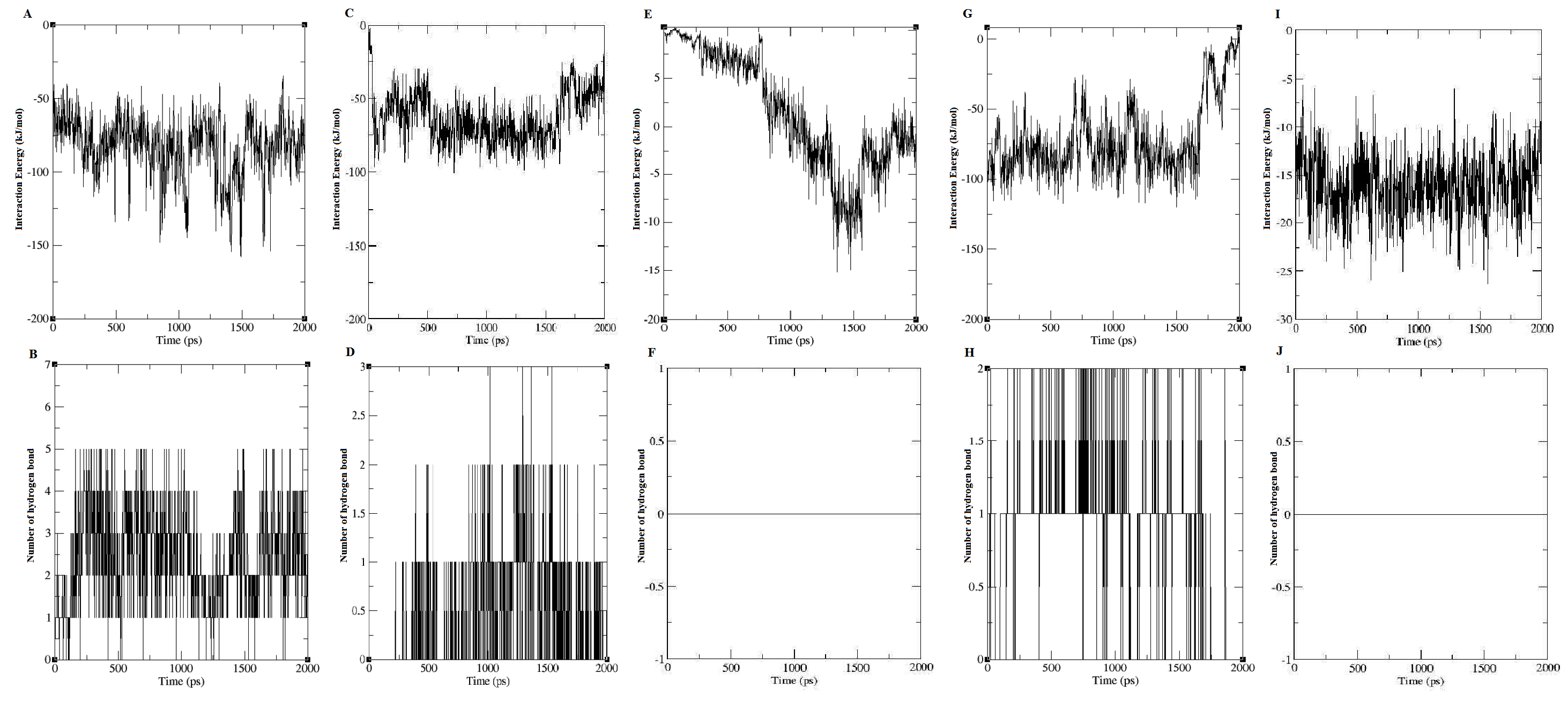

Supplement: S3 Fig — (A), (C), (E), (G) and (I) represent the interaction energy of Compound 3 (Dihydrochalcones), Neq42, Neq175,4p (Thiazolidinones) and 4k (Thiazolidinones), respectively; whereas, (B), (D), (F), (H) and (J) represent the number of hydrogen bonds of the Compound 3 (Dihydrochalcones), Neq42, Neq175, 4p (Thiazolidinones) and 4k (Thiazolidinones), respectively, against SmCL1 during 2 ns simulation. (TIF) [file pone.0123996.s003.tif]
