# Supplementary material for: Novel Non-Peptide Inhibitors against SmCL1 of Schistosoma mansoni: In Silico Elucidation, Implications and Evaluation via Knowledge Based Drug Discovery
Source: PLoS One. 2015 May 1;10(5):e0123996. doi: 10.1371/journal.pone.0123996 (PMC4416924; doi:10.1371/journal.pone.0123996)
Supplement: S1 Table — (PDF) [file pone.0123996.s004.pdf]

Table S1. Ligand structure of best docks from ligand library prepared from ZINC Database.

Ligand/Zinc ID	Ligand Structure
ZINC_1225898	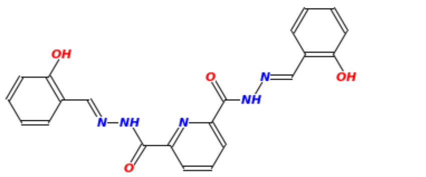
ZINC_43071156	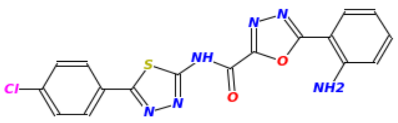
ZINC_8691187	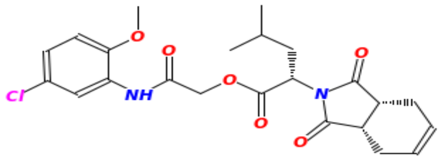
ZINC_20191037	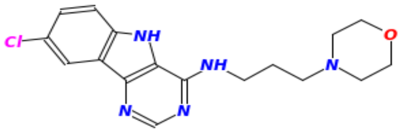
ZINC_6094108	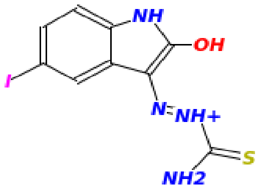

ZINC_46442	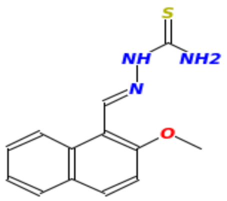
ZINC_17953024	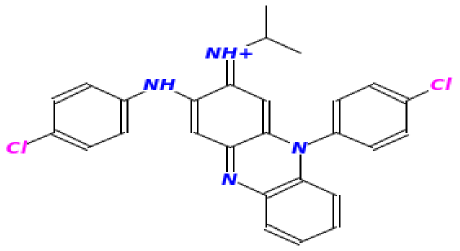
ZINC_344779	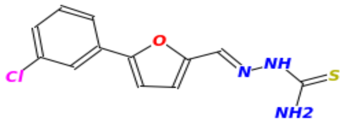
ZINC_22001688	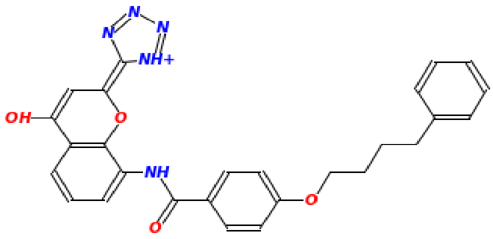
ZINC_13474224	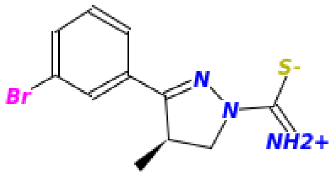

ZINC_2207043	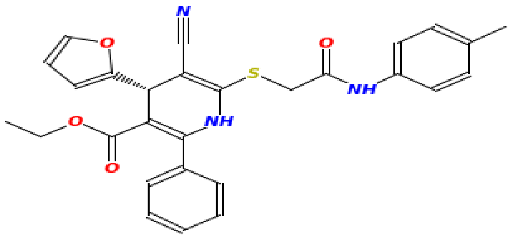
ZINC_1228042	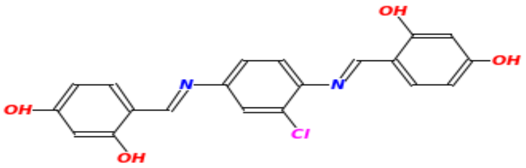
ZINC_12403599	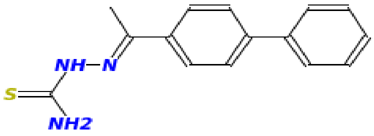
ZINC_13580132	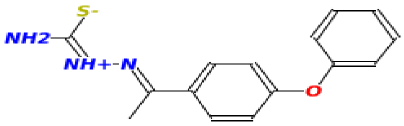
