# Supplementary material for: Novel Non-Peptide Inhibitors against SmCL1 of Schistosoma mansoni: In Silico Elucidation, Implications and Evaluation via Knowledge Based Drug Discovery
Source: PLoS One. 2015 May 1;10(5):e0123996. doi: 10.1371/journal.pone.0123996 (PMC4416924; doi:10.1371/journal.pone.0123996)
Supplement: S2 Table — (PDF) [file pone.0123996.s005.pdf]

Table S2. Ligand structure of best docks from ligand library prepared from Phytochemical Database.

Ligand/PubChem ID	Ligand Structure
CID 5458457	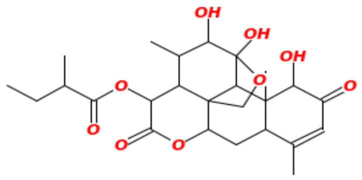
CID 91503	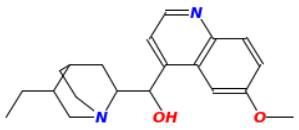
CID 92766	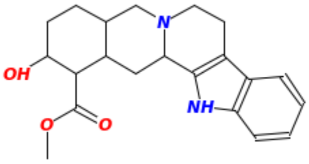
CID 44576034	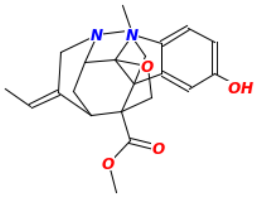
CID 6711208	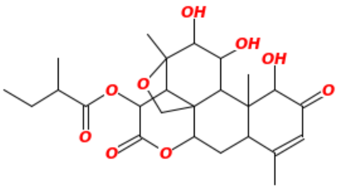

CID 9851692	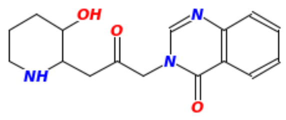
CID 173866	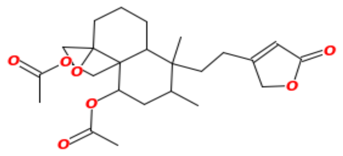
CID 5318998	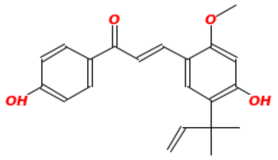
CID 5280961	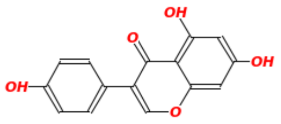
CID 73255	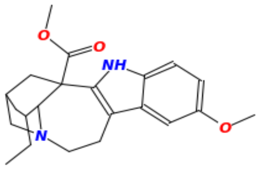

CID 162464	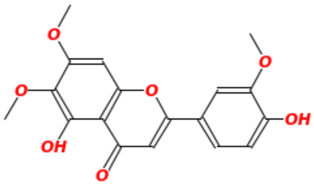
CID 12304613	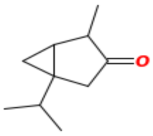
CID 97214	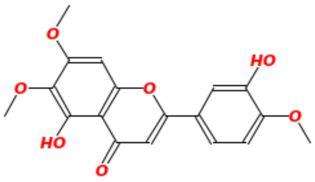
