# Supplementary material for: Novel Non-Peptide Inhibitors against SmCL1 of Schistosoma mansoni: In Silico Elucidation, Implications and Evaluation via Knowledge Based Drug Discovery
Source: PLoS One. 2015 May 1;10(5):e0123996. doi: 10.1371/journal.pone.0123996 (PMC4416924; doi:10.1371/journal.pone.0123996)
Supplement: S3 Table — (PDF) [file pone.0123996.s006.pdf]

Table S3. AutoDock results (binding energy and inhibition constant) and reported inhibition constant (Ki) of peptide based inhibitors against SmCL1.

**Peptide inhibitor**	**AutoDock Binding energy (Kcal/mol)**	**AutoDock Inhibition constant (Ki)**	**Reported Inhibition constant (Ki)**
Mu-Y-(O-methyl)-hF-FMK	-4.91	251.93 µM	3.0×10^{-10}M
Mu-bsu-hF-FMK	-4.73	343.05 µM	1.7×10^{-9} M
Mu-F-hF-FMK	-4.52	484.49 µM	5.5×10^{-9} M
Z-F-R-FMK	-4.41	572.72 µM	9.6×10^{-9} M
Mu-L-hF-FMK	-4.44	588.49 µM	9.8×10^{-9} M
Z-F-A-FMK	-4.18	692.07 µM	2.7×10^{-8} M
Mu-F-nitroR-FMK	-3.88	1.58 mM	1.7×10^{-7} M
Z-F-S(Obz)-CH_2OCO(2,6-Me_2-4-COOMe)Ph	-4.82	288.47 µM	1.4×10^{-9} M
Z-F-K-CH_2OCO(2,4,6-Me_3)Ph	-4.71	358.25 µM	1.8×10^{-9} M
Z-F-A-CH_2OCO(2,6-$(CF_3)_2$)Ph	-4.64	389.27 µM	2.3×10^{-9} M
Z-F-A-CH_2OCO(2,6-Me_2-4-COOMe)Ph	-4.07	757.47 µM	3.3×10^{-8} M
Z-F-A-CH_2OCO(2,4,6-Me_3)Ph	-3.32	2.63 mM	1.4×10^{-6} M
Z-Phe-Ala-DMK	-3.72	1.76 mM	0.1 µM
Z-Phe-Phe-DMK	-3.14	4.77 mM	5 µM
K11777	-3.95	882.77 µM	Not Determined
