# Supplementary material for: Novel Non-Peptide Inhibitors against SmCL1 of Schistosoma mansoni: In Silico Elucidation, Implications and Evaluation via Knowledge Based Drug Discovery
Source: PLoS One. 2015 May 1;10(5):e0123996. doi: 10.1371/journal.pone.0123996 (PMC4416924; doi:10.1371/journal.pone.0123996)
Supplement: S4 Table — (PDF) [file pone.0123996.s007.pdf]

Table S4. AutoDock results (binding energy and inhibition constant) of non-peptide cruzain inhibitors docked with SmCL1 and reported inhibition parameters of cruzain.

**Inhibitor**	**AutoDock Binding energy (Kcal/mol)**	**AutoDock Inhibition constant (Ki)**	**Reported inhibition parameter of cruzain**
**Dihydrochalcones** (Compound 1)	-7.57	2.84 µM	7.1µM (IC50)
**Dihydrochalcones** (Compound 4)	-7.4	3.78 µM	8.7µM (IC50)
**Dihydrochalcones** (Compound 3)	-7.37	3.99 µM	12µM (IC50)
**Dihydrochalcones** (Compound 2)	-7.35	4.11 µM	21.6µM (IC50)
**Nequimed** (Neq 42)	-6.67	13.1 µM	21.6µM (IC50)
**Nequimed** (Neq 165)	-6.48	17.85 µM	31.3µM (IC50)
**Nequimed** (Neq 177)	-6.19	29.16 µM	48.9µM (IC50)
**Nequimed** (Neq 176)	-5.94	44.31 µM	68.5µM (IC50)
**Nequimed** (Neq 172)	-5.8	55.64 µM	73.1µM (IC50)
**Nequimed** (Neq 179)	-3.42	1.52 mM	Inactive
**Nequimed** (Neq 148)	-3.52	2.65 mM	Inactive
**Nequimed** (Neq 155)	-3.75	1.79 mM	Inactive
**Nequimed** (Neq 175)	-3.7	1.94 mM	Inactive
**Thiazolidinones** (4p)	-6.56	15.47 µM	94.3 ± 0.9 (% cruzain inhibition)
**Thiazolidinones** (4n)	-6.53	16.27 µM	78 ± 2 (% cruzain inhibition)
**Thiazolidinones** (4a)	-6.45	17.85 µM	64 ± 2 (% cruzain inhibition)
**Thiazolidinones** (4e)	-5.95	43.85 µM	58 ± 5 (% cruzain inhibition)
**Thiazolidinones** (4g)	-5.88	48.91 µM	50.4 ± 0.3 (% cruzain inhibition)
**Thiazolidinones** (4h)	-5.58	73.29 µM	40 ± 13 (% cruzain inhibition)
**Thiazolidinones** (4f)	-5.45	109.14 µM	37 ± 2 (% cruzain inhibition)
**Thiazolidinones** (4d)	-5.19	156.11 µM	36.1 ± 0.6 (% cruzain inhibition)
**Thiazolidinones** (4m)	-5.01	212.1 µM	31 ± 2 (% cruzain inhibition)
**Thiazolidinones** (4i)	-4.95	234.27 µM	25 ± 2 (% cruzain inhibition)
**Thiazolidinones** (4l)	-4.8	304.12 µM	18 ± 2 (% cruzain inhibition)
**Thiazolidinones** (4o)	-4.65	392.61 µM	15 ± 2 (% cruzain inhibition)
**Thiazolidinones** (4j)	-4.59	430.49 µM	13 ± 3 (% cruzain inhibition)
**Thiazolidinones** (4c)	-4.49	513.17 µM	11 ± 2 (% cruzain inhibition)
**Thiazolidinones** (4b)	-4.28	728.77 µM	5 ± 5 (% cruzain inhibition)
**Thiazolidinones** (4k)	-3.87	1.45 mM	Inactive
**Thiosemicarbazone** (Compound 8)	-6.28	24.88 µM	65 (% cruzain inhibition)
**Thiosemicarbazone** (Compound 7)	-6.08	36.3 µM	55 (% cruzain inhibition)
**Thiosemicarbazone** (Compound 11)	-5.85	51.4 µM	50 (% cruzain inhibition)
**Thiosemicarbazone** (Compound 1)	-4.92	247.35 µM	25 (% cruzain inhibition)
**Thiosemicarbazone** (Compound 9)	-4.87	267.64 µM	20 (% cruzain inhibition)
**Thiosemicarbazone** (Compound 10)	-4.42	574.77 µM	10 (% cruzain inhibition)
**Thiosemicarbazone** (Compound 12)	-4.48	523.52 µM	10 (% cruzain inhibition)
**Thiosemicarbazone** (Compound 5)	-4.26	749.24 µM	5 (% cruzain inhibition)
**Thiosemicarbazone** (Compound 2)	-3.61	2.25 mM	Inactive
**Thiosemicarbazone** (Compound 3)	-3.58	2.37 mM	Inactive
